# Molecular and Functional Analysis of Pore-Forming Toxin Monalysin From Entomopathogenic Bacterium *Pseudomonas entomophila*

**DOI:** 10.3389/fimmu.2020.00520

**Published:** 2020-03-27

**Authors:** Saori Nonaka, Emil Salim, Koki Kamiya, Aki Hori, Firzan Nainu, Rangga Meidianto Asri, Ayu Masyita, Takumi Nishiuchi, Shoji Takeuchi, Noriyuki Kodera, Takayuki Kuraishi

**Affiliations:** ^1^Faculty of Pharmacy, Institute of Medical, Pharmaceutical and Health Sciences, Kanazawa University, Kanazawa, Japan; ^2^Faculty of Pharmacy, Universitas Sumatera Utara, Medan, Indonesia; ^3^Kanagawa Institute of Industrial Science and Technology, Kawasaki, Japan; ^4^Graduate School of Science and Technology, Gunma University, Maebashi, Japan; ^5^Faculty of Pharmacy, Universitas Hasanuddin, Makassar, Indonesia; ^6^Institute for Gene Research, Kanazawa University, Kanazawa, Japan; ^7^Department of Mechano-Informatics, Graduate School of Information Science and Technology, The University of Tokyo, Tokyo, Japan; ^8^WPI Nano Life Science Institute, Kanazawa University, Kanazawa, Japan

**Keywords:** innate immunity, *Drosophila*, pore-forming toxin, atomic force microscope, Monalysin

## Abstract

*Pseudomonas entomophila* is a highly pathogenic bacterium that infects insects. It is also used as a suitable model pathogen to analyze *Drosophila's* innate immunity. *P. entomophila's* virulence is largely derived from Monalysin, a β-barrel pore-forming toxin that damages *Drosophila* tissues, inducing necrotic cell death. Here we report the first and efficient purification of endogenous Monalysin and its characterization. Monalysin is successfully purified as a pro-form, and trypsin treatment results in a cleaved mature form of purified Monalysin which kills *Drosophila* cell lines and adult flies. Electrophysiological measurement of Monalysin in a lipid membrane with an on-chip device confirms that Monalysin forms a pore, in a cleavage-dependent manner. This analysis also provides a pore-size estimate of Monalysin using current amplitude for a single pore and suggests lipid preferences for the insertion. Atomic Force Microscope (AFM) analysis displays its structure in a solution and shows that active-Monalysin is stable and composed of an 8-mer complex; this observation is consistent with mass spectrometry data. AFM analysis also shows the 8-mer structure of active-Monalysin in a lipid bilayer, and real-time imaging demonstrates the moment at which Monalysin is inserted into the lipid membrane. These results collectively suggest that endogenous Monalysin is indeed a pore-forming toxin composed of a rigid structure before pore formation in the lipid membrane. The endogenous Monalysin characterized in this study could be a desirable tool for analyzing host defense mechanisms against entomopathogenic bacteria producing damage-inducing toxins.

## Introduction

The innate immune system is the front line of defense against microbial infection in metazoan animals (1). Innate immune cells can sense infectious threats either by pathogen-associated molecular patterns (PAMPs) or damage-associated molecular patterns (DAMPs) (1–3): pathogen-specific molecules such as peptidoglycans utilized by microbes as an essential substance of their life (1), or host-derived molecules that are normally kept inside in their cells but released because of the tissue damage by infection (2), respectively. The recognition and signaling mechanisms involving PAMPs are relatively well-studied. In *Drosophila*, humoral innate immunity relies on distinct signaling pathways, the Toll pathway and immune deficiency (IMD) pathway (4, 5). The Toll pathway is responsible for infectious threats from fungi or Gram-positive bacteria, and it senses fungal β-glucans or bacterial Lysine (Lys)-type peptidoglycans with a pattern recognition receptor PGRP-SA/GNBP1 complex or GNBP3 in the hemolymph (6–9). PAMPs recognition by those receptors stimulates serine protease cascades in the hemolymph, which produces a cleaved form of the cytokine-like protein Spätzle (Spz), a ligand of a Toll receptor (10). An activated Toll receptor transmits a signal to NF-κB Dif and/or Dorsal through a dMyd88-Tube-Pelle complex, producing antimicrobial factors such as the antifungal peptide Drosomycin (11, 12). The IMD pathway is another NF-κB pathway that recognizes Diaminopimelic acid (DAP)-type peptidoglycans released from Gram-negative bacteria (13), eventually triggering the translocation of Relish to the nucleus and inducing the expression of genes that encode antimicrobial proteins, including Diptericin (14). In contrast to a PAMPs-initiated innate immunity, DAMPs-mediated innate immune mechanisms in terms of an infectious situation have not been well-characterized yet. In flies, protease cascade upstream of a Toll receptor is partly involved in DAMPs recognition. For example, fungal proteases could potentiate the serine protease cascade through Persephone (15–17). Additionally, entomopathogenic nematodes damage epithelial cells and/or cuticles and degrade basement membrane (BM). Clotting and components from disrupted BM seem to have a protective function against nematode infection (18, 19). However, the whole picture of damage-induced innate immunity is far from understood.

*Pseudomonas entomophila* is an entomopathogenic, Gram-negative bacterium that was originally isolated from a wild fly sampled in Guadeloupe in the Caribbean (20). *P. entomophila* displays pathogenicity by oral infection, and the bacteria are widely used as a tool to examine gut innate immune responses (20). Recently, *P. entomophila* has also been used in a systemic infection model (21). In the gut, *P. entomophila* infection imposes severe damage via a reactive oxygen species, produced by host cells and a pore-forming toxin (PFT) from the bacteria, generally inhibiting translation in the intestine and thus blocking epithelium renewal (22). The virulence of *P. entomophila* is under the control of a GacS/GacA two component system (20). One of the main effector molecules in this system is a PFT, Monalysin. Monalysin is secreted as a pro-toxin that is cleaved by proteases, such as AprA in *P. entomophila*, to become fully active (23). *Drosophila* adults combat these effectors using a cross-linked drosocrystallin (dcy) protein, which works as physical barrier blocking the permeation of macromolecules (>500 kDa) in the peritrophic matrix (24, 25). Cleaved Monalysin shows cytotoxic activity, probably by forming pores in the plasma membrane of host cells, leading to disrupted membrane permeability and cell death (23). The secondary structure prediction of the membrane-spanning domain indicates that Monalysin is a PFT of the β type (23). Leone et al. reported that X-ray crystallography and cryo-electron microscopy, with recombinant Monalysin produced in *E. coli*, revealed its 3D structure and its putative mechanism during pore formation in the lipid membrane (26). The recombinant Monalysin is an 18-mer complex composed of two disk-shaped nonamers held together by the N-terminal swapping of the pro-peptides. The membrane-spanning region of pro-Monalysin is fully buried in the center of the ring or torus, and, perhaps during activation upon cleavage, the two disk-shaped nonamers dissociate to leave the transmembrane segments that attach to the target membrane, undergo conformational changes, and form the pore.

In order to study the interaction between the host and entomopathogenic bacteria producing damage-inducing toxins, well-characterized purified Monalysin may be a useful tool. Besides, pore-forming proteins such as Monalysin may potentially be developed as biological control agents against insects (e.g., Cry toxin) (27–29), as well as biological “nanopores” that are used as a detector for single-molecule (e.g., α-hemolysin) (30). In that sense, endogenous Monalysin purified from *P. entomophila* could provide more precise insight of its protein function, rather than using the recombinant protein generated by *E. coli* that may have distinct intracellular environment from *P. entomophila*, which potentially gives rise to a different subunit composition of the protein and thereby could influence the structural and functional features of the molecule. Additionally, a detailed analysis of the structure of the native pore-forming protein and its dynamics in solution and in lipid membrane would serve basic information for various applications. In this study, we succeeded in purifying native endogenous Monalysin from *P. entomophila* with killing activity in *Drosophila* cell line and adult flies. We also characterized its structure and function using electrophysiological measurements and a high-speed atomic force microscope (HS-AFM).

## Materials and Methods

### Bacteria Stocks, Other Materials, and Cell Culture

*P. entomophila* wild-type strain L48 and a Monalysin mutant *mnl* were kindly provided by Dr. B. Lemaitre. 1,2-Dioleoyl-*sn*-glycero-3-phosphocholine (DOPC), 1,2-dioleoyl-*sn*-glycero-3-phospho-L-serine (DOPS), 1,2-dioleoyl-*sn*-glycero-3-phosphoethanolamine (DOPE), and 1,2-dioleoyl-*sn*-glycero-3-phosphoethanolamine-*N*-(cap-biotinyl) (biotin-cap-DOPE) were purchased from Avanti Polar Lipids. n-decane was purchased from Sigma-Aldrich. S2 cells from *Drosophila* hemocytes were maintained at 25°C in Schneider's *Drosophila* medium (Thermo Fisher SCIENTIFIC) containing 10% (v/v) heat-inactivated FBS, 100 units/mL penicillin, and 100 μg/mL streptomycin.

### Purification of Pro-Monalysin

*P. entomophila* was grown in LB at 29°C overnight, and was collected by centrifugation at 8,700 × g at 4°C for 15 min. The cell pellets were washed with PBS and lysed in PBS containing 2% (w/v) CHAPS. Cells were sonicated at 4°C overnight, filtrated with a 70 μm Cell Strainer (BD Falcon), and centrifuged at 9,000 × g at 4°C for 20 min to remove insoluble pellets. The collected supernatant was diluted 10 times with PBS, filtrated with a 0.22 μm filter (Corning), and dialyzed with PBS for 9 h to exchange the solvent. Total lysate (*P. entomophila* extracts) was performed by ammonium sulfate precipitation (25–50%). The pellet was dissolved in 20 mM Tris-HCl, pH8.0 and dialyzed against the same buffer for 9 h to remove salts. The dialysate was then subjected to anion exchange chromatography with a HiTrap Q HP column (GE Healthcare), pre-equilibrated in 20 mM Tris-HCl, pH8.0 and then eluted with a linear gradient 0 to 1 M NaCl dissolved in 20 mM Tris-HCl, pH8.0 at a flow rate of 1 mL/min for 30 min. After a cell viability assay, the fractions with cytotoxic activity were harvested and concentrated by ammonium precipitation (50%). The pellet was dissolved in 10 mM sodium phosphate buffer, pH 7.4, containing 140 mM NaCl, and then subjected to gel filtration chromatography with a Superdex 200 Increase 10/300 GL column (GE Healthcare), pre-equilibrated in a 10 mM Sodium phosphate buffer, pH 7.4, containing 140 mM NaCl, and then eluted with the same buffer at a flow rate of 0.75 mL/min. The peak eluted at 13–14 min (molecular weight: around 460 kDa) was collected and analyzed by SDS-PAGE. The gel was stained with Coomassie Brilliant Blue (Kanto Chemical Co., Inc.) to check the purity. The 30 kDa band was excised and analyzed by mass spectrometry. The molecular weight of each peak in gel filtration chromatography was estimated by loading Gel filtration Calibration Kit HMW (GE Healthcare) in the same column. Protein concentrations of fractions were measured by a Lowry method with Bio-Rad DC protein assay kit (Bio-Rad). In the trypsin treatment to get active-Monalysin, trypsin was added to purified pro-Monalysin (175 μg) at 0.2 mg/mL and incubated at 25°C for 10 min, followed by a Protease Inhibitor Cocktail for General Use (nacalai tesque, Cat# 04080-11) was added. To completely degrade Monalysin, trypsin was added to pro-Monalysin (10 μg) at a concentration of 0.2 mg/mL and incubated at 37°C for 58 h.

### Mass Spectrometry

To identify the protein in the cytotoxic fraction, MALDI-TOF MS/MS analysis was performed at the Institute for Gene Research, Advanced Science Research Center, Kanazawa University, using a tandem mass spectrometer (4,800 plus MALDI TOF/TOF™ Analyzer [Sciex]) with 2,5-dihydroxybenzoic acid (DHB) as a matrix as described in Asano and Nishiuchi (31). Briefly, a cytotoxic fraction was loaded on an SDS-acrylamide gel, and a 30 kDa band was excised and in-gel digested with trypsin. The digested peptides were analyzed by MALDI-TOF/TOF. The data was subjected to the Protein Pilot ver.4.0 (Sciex) against the *Pseudomonas entomophila* (NCBI, Tax ID 312306) protein database (2017-8-23). To determine the molecular weight of the active-Monalysin multimer, MALDI-TOF analysis was performed using the UltrafleXtreme MALDI TOF/TOF Analyzer (Bruker Daltonix) at Fukui Prefectural University with sinapic acid (SA) as a matrix. First, areas on the MALDI plates were coated with the SA solution. Then, the mixture of active-Monalysin with SA was dropped onto the SA-coated spots. Each spot was analyzed to obtain the molecular weight by MALDI-TOF (ultrafleXtreme). The results from several measurements were integrated via analysis software version 4.1.2.

### Cell Viability Assay

S2 cells (1.5–8.0 × 10^5^ cell in 100 μL) were inoculated in a 96-well plate. 10 μL of *P. entomophila* extract or collected fractions after chromatography, purified pro-Monalysin (1.5 μg/mL), active-Monalysin (1.5 μg/mL), or trypsin (3.8 × 10^−2^ μg/mL) were added and incubated at 25°C for 12–18 h. Cell viability was monitored by luminescence from a CellTiter-Glo Luminescent Cell Viability Assay (Promega) with a Spark 10 M (TECAN). Cell viability is expressed as a relative value, with luminescence in cells incubated with the buffer (negative control) being 100%. To measure total activity, cell viability, after incubation with serial diluted fractions, was examined and total activity was calculated as 1 unit corresponding to activity that yields 70% cell viability. Specific activity was expressed as total activity divided by total protein (mg).

### Caspase-3/7 Activity Assay

S2 cells (1.5 × 10^5^ cell in 100 μL) were inoculated onto a 96-well plate. Cycloheximide and active-Monalysin were added at 1.5 μg/mL and incubated at 25°C for 6, 12, 18, and 24 h. Caspase-3/7 activity was monitored by luminescence from a Caspase-Glo 3/7 Assay (Promega) using a Synergy HTX (BioTek).

### Monalysin Injection and Survival Assay

Oregon R flies (*Drosophila melanogaster*, females, 3–7 days after eclosion) were injected with a pro-Monalysin, active-Monalysin, or degraded-Monalysin solution (1 mg/mL) into their hemolymph by micro-injection (70 nL per fly), and kept at 25°C. Surviving flies were counted at 1 h after injection. For dose-dependent analysis, flies were injected with active-Monalysin solution (3–30 μg/mL), and surviving flies were monitored every 12 h for 60 h.

### Total RNA Extraction and Real-Time PCR

Oregon R flies (*Drosophila melanogaster*, female, 3–7 days after eclosion) were injected with an active-Monalysin, degraded-Monalysin solution (50 μg/mL), or 1,000 times dilution of heat-killed *E. coli* into their hemolymph and kept at 25°C for 3, 6, 20 h. To obtain the heat-killed *E. coli*, overnight culture of *E. coli* (DH5α) without dilution were heated at 100°C for 30 min, sonicated for 10 min, and then diluted with water. To quantify the *Drosomycin* (*Drs*), total RNA of the collected flies was isolated with Sepasol-RNA I Super G (nacalai tesque) and used for cDNA synthesis with ReverTra Ace reverse transcriptase (TOYOBO) and oligo (dT)12–18 primers. To quantify the *Diptericin* (*Dpt*), *puckered* (*puc*), and *Turandot A* (*TotA*), isolated RNA were subjected to DNase treatment (Promega, M6101), followed by cDNA synthesis with ReverTra Ace reverse transcriptase (TOYOBO) and oligo (dT)12–18 primers. Quantitative real-time PCR (RT-qPCR) was performed using a LightCycler 480 (Roche Diagnostics). *rpL32* was used as an internal control. The following primers were used for RT-qPCR: *Drs* forward, TTGTTCGCCCTCTTCGCTGTCCT; *Drs* reverse, GCATCCTTCGCACCAGCACTTCA; *Dpt* forward, GTTCACCATTGCCGTCGCCTTAC; *Dpt* reverse, CCCAAGTGCTGTCCATATCCTCC; *puc* forward, GGCCTACAAGCTGGTGAAAG; *puc* reverse, AGTTCAGATTGGGCGAGATG; *TotA* forward, CCAAAATGAATTCTTCAACTGCT; *TotA* reverse, GAATAGCCCATGCATAGAGGAC; *rpL32* forward, AGATCGTGAAGAAGCGCACCAAG; *rpL32* reverse, CACCAGGAACTTCTTGAATCCGG.

### Immunohistochemistry

For oral ingestion of Monalysin, *dcy*^1^ flies (Bloomington #26106, females, 3–7 days after eclosion) obtained from the Bloomington *Drosophila* Stock Center were starved for 2 h at 29°C, then placed in a fly vial with the food solution. The food solution consisted in a mixture of active-Monalysin solution (4 mg/mL) and 5 % sucrose (1:1), which was added to a filter disk that completely covered the surface of the standard fly medium. Flies were kept at 29°C for 8 h, after which their guts were dissected out. Antibody staining was performed as previously described by Kenmoku et al. (32) with 1:200 rabbit anti-PH3 (Cell Signaling, Cat #9701), 1:50 mouse anti-Dlg (Developmental Studies Hybridoma Bank), and 1:200 Alexa 555-coupled and Alexa 488-coupled secondary antibodies (Thermo Fisher SCIENTIFIC). Nuclei were stained by 0.1 μg/mL of 4',6-diamidino-2-phenylindole (DAPI). Samples were visualized with a LSM710 confocal microscope (Carl Zeiss) or observed using a conventional fluorescent microscope and images were reconstructed using Photoshop (Adobe).

### SLP Assay for Purified Monalysin

To examine the contamination level of peptidoglycan, 10 μL of 0.001–1 mg/mL pro-Monalysin, active-Monalysin and degraded Monalyin were incubated with 40 μL of Silkworm Larvae Plasma (SLP) reagent (Wako) at 25°C for 30 min in a 96-well plate. The SLP reagent contains all factors involved in the prophenoloxidase cascade system triggered by peptidoglycans, which consequently activates prophenoloxidase. The activated prophenoloxidase then oxidizes 3,4-dihydroxyphenylalanine (DOPA) in the substrate, thus forming a black melanin pigment. The amount of peptidoglycan was monitored as the blackness of the mixture visually. As a positive control, several dilution (1/10, 1/10^2^, 1/10^3^, 1/10^4^) of heat-killed *E. coli* solution were subjected to the same test.

### Ion Current Measurement of Monalysin Using a Bilayer Lipid Membrane (BLM) Chip With 16 Separate Channels

One microliter of pro-Monalysin solution (1.5 mg/mL) was added to 0.1 μL of trypsin (0.25 % [w/v]). The mixture was incubated for 10 min at room temperature (~23°C). To form a planar BLM, using the droplet contact method; 3.7 μl of lipid dissolved in *n*-decane (20 mg/mL dioleoylphosphatidylcholine [DOPC] or dioleoylphosphatidylcholine, dioleoylphosphatidylserine, and dioleoylphatidylethanolamine [DOPC/DOPS/DOPE] [molar ratio of 7:2:1]) was added to each double well on a BLM chip with 16 separate channels (16-ch). Twenty one microliter of buffer solution (20 mM Tris-HCl/ 150 mM NaCl [pH 8.8]) containing 0.015 mg/mL of Monalysin solution was added to each double well. The planar BLM was formed at the macroapertures. The Monalysin's current signals were recorded using a multichannel patch clamp amplifier with a 1-kHz low-pass filter at a sampling frequency of 5 kHz (Tecella JET). The measurement temperature was 23 ± 1°C. Current analysis was performed using the pCLAMP software program (molecular devices).

### Atomic Force Microscopy

AFM imaging was performed in a solution at room temperature (24–26°C), using a laboratory-built high-speed AFM setup (33) as described in Uchihashi et al. (34). For AFM substrates, two types were used: the flat muscovite mica substrate and the polydimethylsiloxane (PDMS) substrate with controlled convex shapes (ca. 50 nm) (35). Either a mica disc (1.5 mm in diameter and ~0.05 mm in thickness) or a PDMS disk (2 mm in diameter and ~0.02 mm in thickness) was glued on a glass sample stage (2 mm in diameter and height) by epoxy. A freshly cleaved mica surface was prepared by removing the top layers of mica using Scotch tape. The PDMS surface was hydrophilized by a plasma ion bomber (PIB-10, Vacuum Device) set to hard mode for 3 min. The glass stage with either substrate was attached to the top of a Z-scanner by a drop of nail polish, on which a drop (2 μL) of sample solution (either 0.1 mg/mL Monalysin or 0.1 mg/mL liposome) was deposited. The liposome solution was prepared as previously described (34), and the lipid composition was DOPC:DOPS:biotin-cap-DOPE = 7:2:1 (w/w). After incubation for 3–5 min, the substrate surface was rinsed with 20 μL of the observation buffer to remove floating samples. The sample stage was then immersed in a liquid cell containing ~60 μL of the observation buffer. AFM imaging was carried out in tapping mode, using small cantilevers (BLAC10DS-A2, Olympus), with a resonant frequency of ~0.5 MHz in water, a quality factor of ~1.3 in water, and a spring constant of ~0.08 N/m. The cantilever's free oscillation amplitude *A*_0_ and set-point amplitude *A*_s_ were set at 1–2 nm and ~0.9 × *A*_0_, respectively. In some experiments, high tapping forces were applied to the samples by reducing *A*_s_, and a protein solution containing either Monalysin or trypsin (5 μL) was injected in the observation buffer during high-speed atomic force microscopy (HS-AFM) imaging. The imaging rate, scan size, and the pixel size for each AFM image are described in figure legends.

### Analysis of AFM Images

AFM images were pretreated for analysis by a low-pass filter to remove spike noise and a flatten filter to make the overall xy-plane flat, using a laboratory built software as described in Ngo et al. (36). The molecule heights were measured semi-automatically using the following steps. First, the most probable highest point near the highest point of the molecule was selected manually. Second, the actual highest point was determined automatically by searching a 10 × 10-pixel area (typically 10 × 10 nm^2^) around the selected point. The surface area occupied by the specific molecular species was analyzed by ImageJ using binarized images. The binarized images were obtained by setting a threshold height. The threshold heights were 8 nm for the double-ring complex of pro-Monalysin and 4 nm for the single-ring complex of pro- and active-Monalysin, respectively.

### Statistical Analysis

Statistical analyses were performed by a Student's *t*-test or log-rank test, and *P* < 0.05 were considered significant.

## Results

### Purification of Endogenous Monalysin Protein From *P. entomophila*

**Figure 1:**
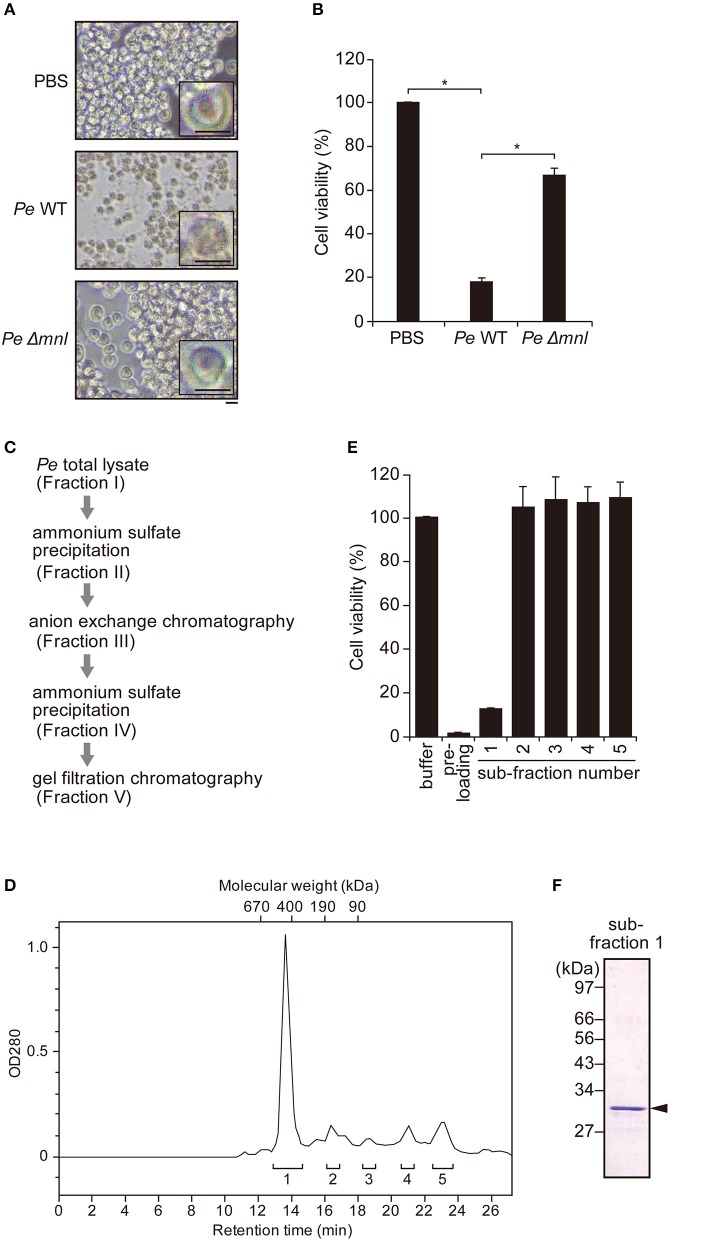
Purification of endogenous pro-Monalysin from *P. entomophila*. **(A)** Phase contrast images of S2 cells after incubation with PBS, wild type (*Pe* WT) or Monalysin-deficient strain (*Pe* Δ*mnl*) of *P. entomophila* extracts (15 μg protein/8 × 10^5^ cells in 100 μL) for 12 h. Magnification images are shown in the square on the right side. Scale bar; 20 μm. The space around the S2 cells after incubation with *Pe* WT extracts appears whiter than others, due to cell debris. **(B)** S2 cells were incubated with *Pe* WT or *Pe* Δ*mnl* total lysates (15 μg protein/8 × 10^5^ cells in 100 μL) for 12 h. Cell viability was monitored as luminescence by a CellTiter-Glo Luminescent Cell Viability Assay. Cell viability is shown relative to luminescence in cells that were incubated with PBS, taken as 100%. The means ± S.E. obtained with the data from triplicate samples, are presented (*P < 0.05, as determined by a Student's *t*-test). **(C)** The purification step of endogenous pro-Monalysin. A HiTrap Q HP column and a Superdex 200 Increased 10/30 L GL column were used in anion exchange chromatography and gel filtration chromatography, respectively. **(D)** Chromatogram of gel filtration chromatography. Eluted proteins were detected by measuring OD_280_. The retention time was the time passed after loading the sample into the column. The molecular weight of each retention time was estimated by loading Gel filtration Calibration Kit HMW (GE Healthcare) in the same column. The estimated molecular mass was around 460 kDa for the first eluted peak. Pre-loading indicates a fraction before loading to column for gel filtration chromatography (that is, it is the same Fraction IV in Table 1). Brackets and numbers were collected fractions and sub-fraction numbers, respectively. **(E)** S2 cells were incubated with each fraction obtained from gel filtration chromatography for 12 h. Cell viability was monitored as luminescence by a CellTiter-Glo Luminescent Cell Viability Assay. Cell viability is shown relative to luminescence in cells incubated with an elution buffer, taken as 100%. The means ± S.E. obtained with the data from triplicate samples, are presented. **(F)** SDS-PAGE analysis of fraction 1. The gel was stained with Coomassie Brilliant Blue. The arrowhead indicates a pro-Monalysin monomer (30 kDa). The numbers on the left side indicate molecular weight.

In our previous study, we demonstrated that an extract from *P. entomophila*, prepared by sonication of the bacterial cells with detergent, followed by membrane-filtration, is fatal to adult flies if ingested (24). To examine whether *P. entomophila* extract acts as source for the purification of endogenous Monalysin, we tested whether *P. entomophila* extract had Monalysin-derived cytotoxic activity. *P. entomophila* extract was simply added to the *Drosophila* embryonic hemocyte-derived S2 cell culture, and we found that, after 12 h of incubation, almost all cells lost their normal morphology and fell apart (Figure 1A). A CellTiter-Glo Luminescent Cell Viability Assay, which measures cellular ATP, indicated that the S2 cells were dying (Figure 1B).

Next, we performed the cytotoxic assay, using an extract from a Monalysin-deficient strain of *P. entomophila* to know whether *P. entomophila* extract-induced cell death depends on Monalysin. We found that an extract from a Monalysin-deficient strain showed less cytotoxicity than that of a wild type (Figures 1A,B). This indicates that a *P. entomophila* extract contains Monalysin toxin, and that endogenous Monalysin could be purified using the extract. Furthermore, we expected that this cytotoxic assay could be used to find fractions containing Monalysin in each purification step. We attempted its purification in this way (Figure 1C, Table 1). First, a *P. entomophila* extract or total lysate were precipitated with ammonium sulfate to reduce the extract volume. The precipitate was suspended with a Tris buffer, subjected to a column for anion exchange chromatography, and eluted by a linear gradient of 0 to 1 M NaCl. The active fraction (Fraction III) was subjected to an ammonium sulfate precipitation again and suspended with a phosphate buffer (Fraction IV). Fraction IV was subjected to a column for gel filtration chromatography, and several sub-fractions were collected. A CellTiter-Glo Luminescent Cell Viability Assay showed that only sub-fraction 1 contained cytotoxic activity (Figure 1E). The recovered activity of this final fraction (Fraction V in Table 1 and Figure 1C, sub-fraction 1 in Figures 1D,E) was 6% that of the starting total lysate of *P. entomophila*. The specific activity increased 14-fold (Table 1). The chromatogram of the final fraction (Fraction V in Table 1 and Figure 1C, sub-fraction 1 in Figures 1D,E) showed a sharp single peak around 460 kDa and gave a single band with a molecular mass of 30 kDa on an SDS-PAGE (Figure 1F), which is the estimated size of the pro-form of Monalysin monomer. Mass spectrometric analysis of the single band resulted in specific amino acid sequences of Monalysin (data not shown). From these results, we concluded that Monalysin, as a pro-form, was purified as a homogeneity from the wild type *P. entomophila*. Note that the molecular mass estimated by gel filtration (460 kDa), that of the multimer of Monalysin, was slightly smaller than the estimate found in previous literature (26).

**Table 1:** Purification of pro-Monalysin.

**Fraction**		**Total protein (mg)**	**Total activity^a^ (units)**	**Specific activity^b^ (units/mg protein)**	**Purification (fold)**	**Yield (%)**
I	Total lysate	3.5	3.1 × 10^3^	8.7 × 10^2^	1.0	100
II	25–50% (NH4)_2_SO_4_ ppt^c^	2.6	1.7 × 10^3^	6.8 × 10^2^	0.78	57
III	Anion exchange HPLC	2.3 × 10^−1^	1.6 × 10^3^	7.0 × 10^3^	8.0	52
IV	0–50% (NH4)_2_SO_4_ ppt	6.0 × 10^−2^	4.7 × 10^2^	8.4 × 10^3^	9.7	16
V	Gel filtration HPLC	2.0 × 10^−2^	1.8 × 10^2^	1.2 × 10^4^	14	6

a *Total activity was calculated as 1 unit corresponding to activity that yields 70% cell viability*.

b *Specific activity indicates total activity divided by total protein*.

c *ppt: precipitate*.

**Figure 2:**
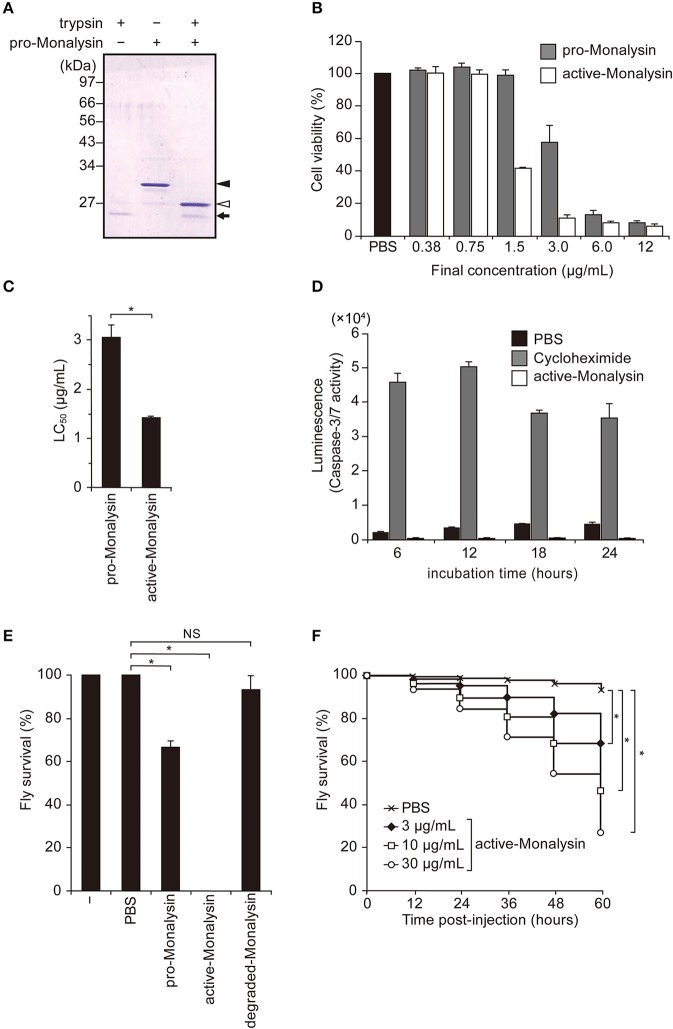
Trypsin treatment transforms purified pro-Monalysin into its active-form. **(A)** A SDS-PAGE analysis of purified pro-Monalysin before and after trypsin treatment. The gel was stained with Coomassie Brilliant Blue. Trypsin was incubated with purified pro-Monalysin at 0.2 mg/mL for 10 min. Closed and open arrowheads indicate a pro-Monalysin monomer (30 kDa) and an active-Monalysin monomer (27 kDa), respectively. The arrow indicates trypsin. **(B)** Cell viability after incubation with pro-Monalysin and active-Monalysin. S2 cells (1.5 × 10^5^ cells in 100 μL) were incubated with the indicated concentration of pro-Monalysin or active-Monalysin for 18 h, cell viability was measured via a CellTiter-Glo Luminescent Cell Viability Assay. Cell viability is shown, relative to luminescence in cells incubated with PBS, taken as 100%. **(C)** LC_50_ of pro-Monalysin and active-Monalysin. LC_50_ was estimated from data in the **B**. The means ± S.E. obtained with the data from triplicate samples are presented (*P < 0.05, as determined by a Student's *t*-test). **(D)** Caspase-3/7 activity in cells after incubation with active-Monalysin. S2 cells (1.5 × 10^5^ cell in 100 μL) were incubated with active-Monalysin or Cycloheximide (an apoptosis inducer) at 1.5 μg/mL for the indicated time. Caspase-3/7 activity was measured as luminescence using a Caspase-Glo 3/7 Assay. The means ± S.E. obtained with the data from duplicate samples in two independent experiments **(E)** Survival analysis of adult flies upon injection with pro-Monalysin, active-Monalysin, or degraded-Monalysin (1 mg/mL) into their hemolymph for 1 h (*P < 0.05; NS, not significant, as determined by a Student's *t*-test). The minus indicates un-injected flies. The means ± S.E. were obtained with the data from three vials (10 flies/each). The data represents two independent experiments**. (F)** Survival analysis of adult flies upon injection with active-Monalysin (3–30 μg/mL) at indicated time points (*P < 0.0001, as determined by a log-rank test).

**Figure 3:**
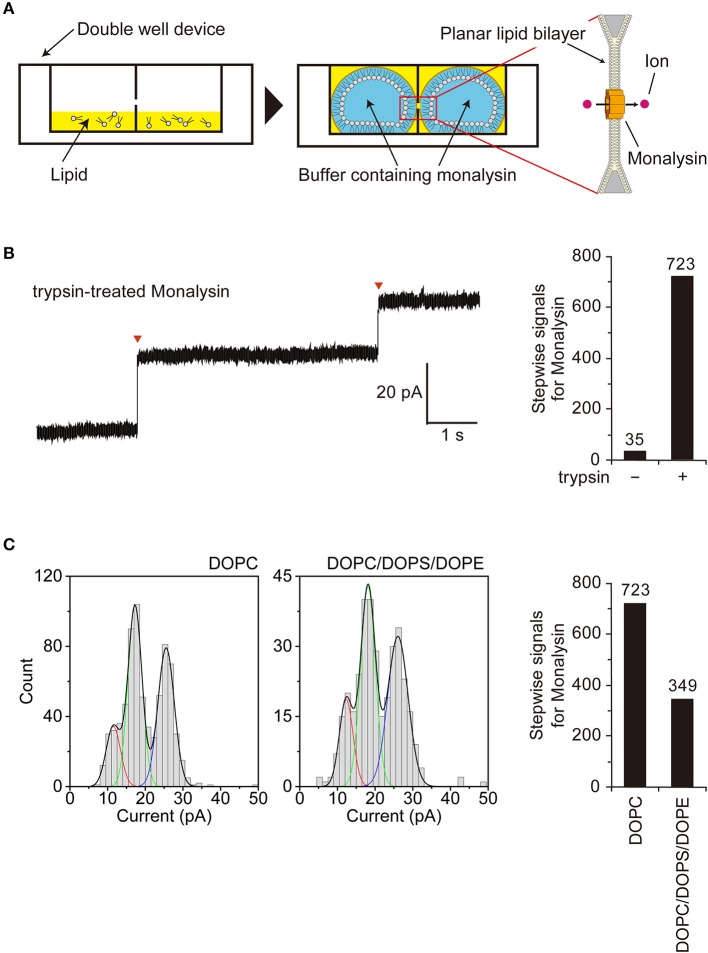
Characterization of Monalysin as a pore-forming toxin using electrophysiological measurements. **(A)** Experimental design for monitoring Monalysin pore formation. A buffer containing Monalysin was supplied to a planar bilayer lipid membrane, prepared by the droplet contact method, and pore formation was monitored by recording ion current signals. **(B)** Typical current trace of the Monalysin, which was digested by trypsin. Applied potential: +100 mV (left). Red triangles represent the detections of a single Monalysin nanopore within the BLM. Total stepwise signals of Monalysin, with or without trypsin treatment, on the DOPC lipid bilayer were shown. Signals for Monalysin were observed for 30 min using a 16-ch device (right). **(C)** Current-amplitude histogram of the Monalysin onto lipid bilayers composed of DOPC and DOPC/DOPS/DOPE (mol ratio of 7:2:1) using a 16-ch device. Applied potential: +100 mV (left). Total signals of Monalysin on the DOPC and DOPC/DOPS/DOPE lipid bilayer were shown. Signals for Monalysin were observed for 30 min using a 16-ch device (right). The curve represents a multipeak Gaussian fitting (*P* < 0.05, *F*-test, respectively): the red, green and blue curves show the first, second and third Gaussian peak, respectively, and the black curves represent the sum of the three Gaussian curves.

Pro-Monalysin is considered to undergo proteolytic cleavage by AprA, a protease secreted by *P. entomophila*, in order to be fully activated as a toxin (23). Leone et al. showed that trypsin cleavage of recombinant pro-Monalysin recapitulated the proteolysis by AprA (26). Thus, we performed trypsin cleavage on our purified endogenous Monalysin to see whether it transforms from a pro-form to an active-form. SDS-PAGE analysis showed that 30 kDa of pro-Monalysin monomer was cleaved to 27 kDa of monomer, as previously reported (Figure 2A). Hereafter, we refer to trypsin-treated endogenous pro-Monalysin as active-Monalysin since the cleaved form exhibited much higher cytotoxic activity than the pro-form (Figure 2B). Note that the trypsin in active-Monalysin did not show cytotoxic activity (Supplementary Figure 1). A lethal concentration of 50% (LC_50_) of pro- and active-Monalysin was estimated from Figure 2B as 1.4 and 3.1 μg/mL, respectively (Figure 2C), which suggested that pro-Monalysin is also toxic to S2 cells. We interpreted this to mean that pro-Monalysin has cytotoxic activity without trypsin treatment because it can undergo proteolysis with some proteases of S2 cells in a cultured medium or on the cell surface, since the pore formation efficiency of pro-Monalysin in artificial membranes is much lower than active-Monalysin (Figure 3B). This cell death induced by active-Monalysin appears to be necrotic rather than apoptotic, as the cells did not show caspase-3/7 activation, while cells treated with cycloheximide (reported to induce typical apoptosis in S2 cells) showed significant induction of the caspase-3/7 activity [(37); Figure 2D]. These results are consistent with cell death induced by Monalysin produced in *E. coli* described in Opota et al. (23).

To confirm whether purified Monalysin had toxic activity *in vivo*, we injected Monalysin into adult hemolymphs. Figure 2E shows that active-Monalysin killed adult flies more efficiently than pro-Monalysin and degraded-Monalysin that is digested by trypsin for a long time to be fully decayed (Supplementary Figure 2), and the insecticidal effect of active-Monalysin has a dose-dependent effect (Figure 2F). Furthermore, the analysis of *dcy* mutant guts fed with active-Monalysin revealed that the number of mitotic stem cells using phospho-histone H3 (PH3) staining (38)—an indicator of gut repair after damage—increased after ingestion of active-Monalysin (Supplementary Figure 3A). In addition, an immunostaining of the septate junction marker Discs large (Dlg) (39) and nuclear staining in flies after oral injection of active-Monalysin showed disrupted organization of the epithelial cells (Supplementary Figure 3B), implying that Monalysin could damage the flies' intestines. Collectively, these results suggest that purified Monalysin has toxic activity *in vivo*.

We also examined whether Monalysin injections could induce antimicrobial peptides (AMP) and stress gene expressions in adult flies, as tissue damage could induce infection-independent humoral innate immunity. Real-time quantitative PCR (RT-qPCR) analysis suggested that the expression of *Drosomycin* (*Drs*) and *Turandot A* (*TotA*), a read-out of the activation of the JAK-STAT pathway, was significantly induced by the injection of active-Monalysin (Supplementary Figures 4A,D). This suggests that Monalysin activates the innate immune and stress pathways, possibly through the Toll and/or JAK-STAT pathways. Monalysin injection, however, did not induce *Diptericin* (*Dpt*) and *puckered* (*puc*) gene expression, a read-out of the activation of the IMD pathway and Jun-N-terminal kinase (JNK) pathway (Supplementary Figures 4B,C). *P. entomophila* is a Gram-negative bacterium which does not contain the Lys-type peptidoglycan recognized by the Toll pathway. In addition, degraded-Monalysin did not induce *Drs* and *TotA* expression (Supplementary Figures 4A,D) and a peptidoglycan-contamination test using Silkworm Larvae Plasma (SLP). Reagent did not show significant contamination of peptidoglycans, which normally activate innate immunity, in active-Monalysin (Supplementary Figures 4E,F). These results might exclude the possibility that contamination of some PAMPs activates humoral immunity and imply that tissue damage induced by Monalysin might induce a humoral innate immune response and stress response in adult *Drosophila*. Taken together, these results show we succeeded to purify endogenous Monalysin, which has a toxic and damage-inducing activity in *Drosophila*.

### Electrophysiological Characterization of Monalysin as a Pore-Forming Toxin

We next sought to confirm that endogenous Monalysin indeed functions as a PFT and characterize its mode-of-action by functional analysis. To monitor pore formation on the lipid membrane, we adopted an “on-chip lipid bilayer system,” which was composed of a parallel ion current recording device with 16 separate channels (16-ch) of artificial planar BLM wells, where the bilayers in the wells were formed based on the droplet contact method (Figure 3A) (40). First, we observed the formation of Monalysin nanopores onto lipid bilayers via the electrophysiological analysis of an artificial cell membrane. We obtained stepwise signals specific to nanopore-containing proteins in the solution containing the active-Monalysin (Figure 3B, left). A total of 723 stepwise signals for active-Monalysin on the DOPC lipid bilayer were observed for 30 min using a 16-ch device (*N* = 2). On the other hand, in case of the pro-Monalysin, 35 stepwise signals were observed for 30 min using a 16-ch device (*N* = 2) (Figure 3B, right). These results suggest that the trypsin-treated active-Monalysins were more vigorously reconstituted into the lipid bilayer and formed nanopores within it.

Next, we investigated the appearance of the active-Monalysin on lipid bilayers composed of DOPC and DOPC/DOPS/DOPE (mol ratio of 7:2:1). The formation of the Monalysin nanopores in the lipid bilayers was more occurrent on the DOPC lipid bilayer (723 stepwise signals) than the DOPC/DOPS/DOPE lipid bilayer (349 stepwise signals) (Figure 3C). We found two amplitude peaks for the active-Monalysin-specific stepwise signals in each case: 1.5 ± 1.9 pA, 17.3 ± 1.9 pA, and 25.5 ± 2.2 pA (mean ± S.D.) for the DOPC lipid bilayer, and 12.3 ± 1.8 pA, 18.1 ± 2.0 pA, and 26.1 ± 2.7 pA (mean ± S.D.) for the DOPC/DOPS/DOPE lipid bilayer (Figure 3C, left). The amplitude peaks of the active-Monalysin signals in case of the DOPC and DOPC/DOPS/DOPE bilayers showed no significant differences. We estimated the diameters of the active-Monalysin nanopores from the amplitude of the active-Monalysin signals and buffer conductance, in accordance with the method described in Gutsmann et al. (41). The diameters of the active-Monalysin nanopores were estimated, using the amplitude peaks, to be 0.74 ± 0.30 nm, 0.91 ± 0.30 nm, and 1.10 ± 0.32 nm (mean ± S.D.) in case of the DOPC lipid bilayer, and 0.77 ± 0.30 nm, 0.77 ± 0.29 nm, 0.93 ± 0.31 nm, and 1.12 ± 0.36 nm (mean ± S.D.) in case of the DOPC/DOPS/DOPE lipid bilayer. In summary, Monalysin appears to insert itself preferably within a lipid bilayer with high ratio of PC, and forms pores measuring around 0.7–1 nm, regardless of the lipid composition.

### Atomic Force Microscope Analysis for the Structure of Monalysin in Solution

Gel filtration chromatography of endogenous Monalysin indicates that Monalysin forms a stable pore-forming complex before activation and membrane interaction, as previously suggested (26). However, based on the gel filtration analysis, it seems that the molecular weight of a Monalysin complex is slightly smaller than that of a previous 18-mer model of Monalysin. Data from an MALDI-TOFMS analysis is in line with this estimate. We detected a possible molecular ion peak of active-Monalysin multimers, whose m/z was 217932.23. This value was very close to the molecular weight of an 8-mer active-complex expected from the amino acid sequences, 213160.4 Da (Supplementary Figure 5). Revealing the structure of native Monalysin in solution and lipid membrane, particularly its dynamic nature, is essential to understand its detailed molecular function and use to evaluate innate immunity mechanisms in flies, as well as to develop biological control agents against insects and biological nanopores. To this end, we employed HS-AFM that enabled dynamic real-time observations of macromolecules at nanometer resolutions, which are not feasible with other methods (42, 43), and had recent achievements of revealing the dynamic structures of pore-forming proteins (44–47).

**Figure 4:**
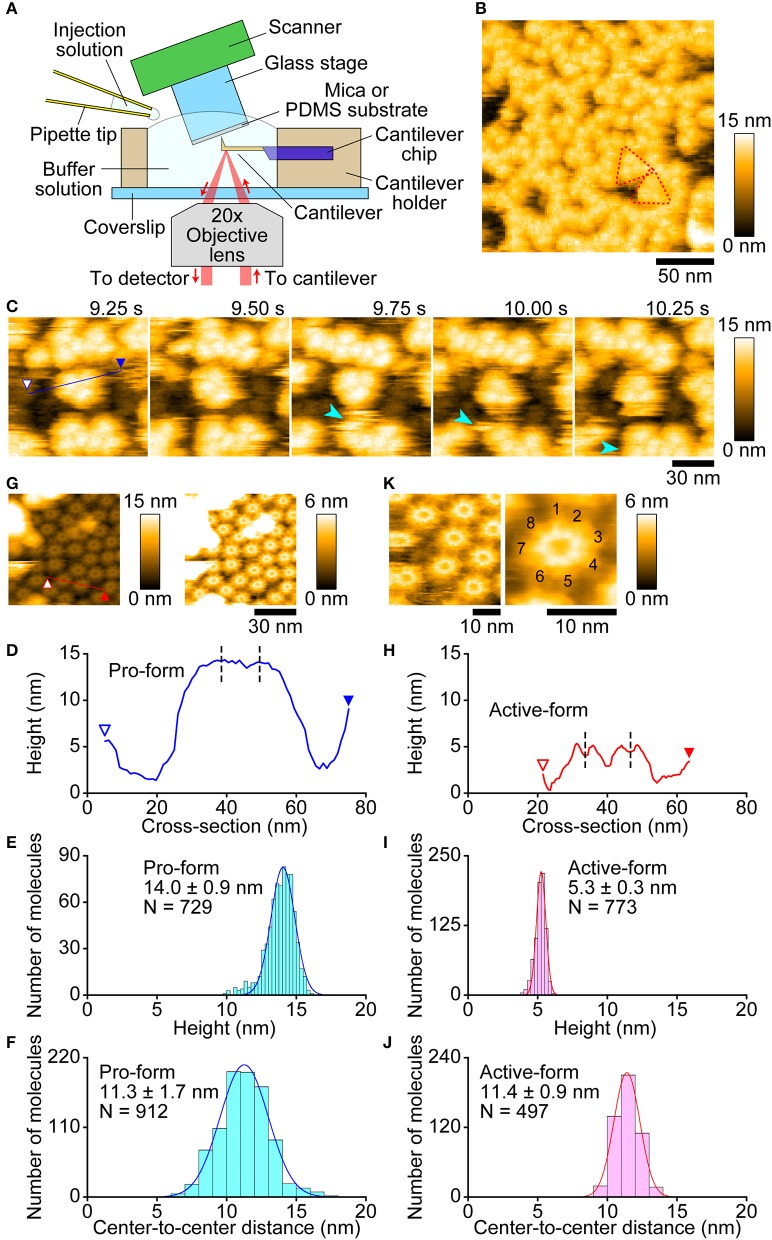
Molecular features of pro- and active-Monalysin on a mica surface visualized by AFM. **(A)** Experimental design for AFM analysis. Samples were absorbed into a substrate surface and imaged by a probe-tip attached at the end of a cantilever. In some experiments, the injection solution was added to the buffer solution during AFM imaging. **(B)** A wide-area image of pro-Monalysin. Typical trefoil-shaped molecules are encircled by red dashed-lines. The scanning area was 200 × 200 nm^2^ with 100 × 100 pixels, and the imaging rate was 330 ms/frame. **(C)** Successive AFM images of pro-Monalysin (see Supplementary Movie 1). The light blue arrowhead shows that a pro-form detaches from, and binds to, a trefoil-shaped molecule. The scanning area was 100 × 100 nm^2^ with 100 × 100 pixels and the imaging rate was 250 ms/frame. **(G)** A wide-area image of active-Monalysin. Two different height scale images are shown. Bright spots are some adsorbed debris. The scanning area was 80 × 80 nm^2^ with 160 × 160 pixels and the imaging rate was 330 ms/frame. **(K)** Small-area image of active-Monalysin (see Supplementary Movie 3). The scanning area was 40 × 40 nm^2^ with 120 × 120 pixels, and the imaging rate was 150 ms/frame. The right image is an averaged image using four successive images. **(D,H)** A cross-section analysis of pro- and active-Monalysin. The sections are from the blue and red lines drawn on the images in **C,G**. Each dashed line indicates the center position of a molecule used in the analysis of **F,J**. **(E,I)** Height distributions of pro- and active-Monalysin. **(F,J)** Center-to-center distance distributions of pro- and active-Monalysin. All distributions were fitted by single-Gaussian curve.

First, we observed pro-Monalysin in the PBS buffer on a mica surface. The experimental setup is shown in Figure 4A. As shown in Figure 4B, molecules with a uniform height covered the mica surface. At smaller scan sizes, trefoil-shaped molecules were seen (Figure 4C, Supplementary Movie 1). Importantly, the molecule corresponding to each leaf of the trefoil dissociated from, and re-bound to, a trefoil-shaped molecule (Figure 4C, 9.75, and 10.25 s), indicating that one particle in the trefoil-shaped molecule is the minimum unit of pro-Monalysin. Note that pro-Monalysin occasionally forms a trimer of the minimum unit in the solution. Hereafter, we refer to this minimum unit of pro-Monalysin as pro-form. The pro-form height was 14.0 ± 0.9 nm (mean ± S.D) (Figures 4D,E). The center-to-center distance between the adjacent pro-forms in the trefoil-shaped molecule was 11.3 ± 1.7 nm (Figure 4F). This result suggests that this distance corresponds to the maximal pro-form diameter, which is slightly smaller than the reported value of recombinant Monalysin (~14 nm) (26). We did not confirm the presence of the reported pore structure on the center of the pro-form. This is because the pro-form moved faster than the AFM scanning speed.

Next, we observed active-Monalysin in the PBS buffer on a mica surface. As shown in Supplementary Figure 6A, no trefoil-shaped molecules were observed, but molecules with a similar width to that of the pro-form were rapidly moving on the mica surface (Supplementary Movie 2). The striking difference from the pro-form was that the height of the molecules was 5.1 ± 0.3 nm (Supplementary Figures 6B,C), which is less than half of the pro-form. This result is consistent with the idea that pro-Monalysin, double-stacked disk-like oligomers, dissociates into two disk-shaped oligomers upon proteolytic cleavage (26). This suggests that we could observe the active-form of Monalysin. However, the presence of the central pore could not be confirmed as the molecules moved rapidly.

Use of a low salt buffer (30 mM NaCl, 10 mM Sodium phosphate, pH 7.0) as an observation buffer induced strong immobilization of Monalysin oligomers on the mica surface, allowing us to image the molecular feature at a high spatial resolution. As shown in Figure 4G, oligomers with a central pore were clearly visualized by the AFM even at somewhat larger scan sizes. The height was 5.3 ± 0.3 nm (mean ± S.D.) (Figures 4H,I), identical to that obtained under the PBS buffer. The central pore had an aperture diameter of ~3 nm (Figure 4H), consistent with the previous report (26). The center-to-center distance between the adjacent active-forms was 11.4 ± 0.9 nm (Figure 4J), indicating that the maximal diameter of the active-form is identical to that of the pro-form. This result is consistent with no significant change induced in the outer diameters of Monalysin upon protease activation (26). At smaller scan sizes, the sub-unit stoichiometry was directly resolved (Figure 4K, Supplementary Movie 3). Unexpectedly, the active-Monalysin was composed of eight sub-units and formed a disk-shaped octamer, in contrast to the crystalline structure of recombinant pro-Monalysin (26), which suggests nonameric (9-mer) composition.

**Figure 5:**
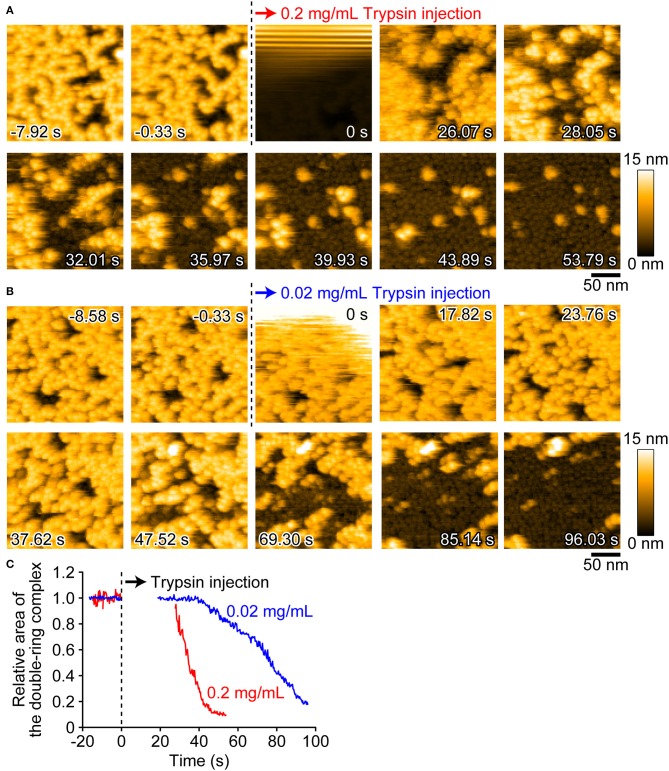
AFM images showing the height conversion of pro-Monalysin upon trypsin treatment. **(A,B)** Successive AFM images before and after trypsin injection (Supplementary Movie 4). The scanning area was 200 × 200 nm^2^ with 100 × 100 pixels, and the imaging rate was 330 ms/frame. At 0 s, a drop of trypsin solution was injected in the observation buffer. The final concentrations of trypsin in the observation buffer were 0.2 mg/mL for **A** and 0.02 mg/mL for **B**, respectively. After injection, the height conversion was gradually seen. **(C)** The time course of the relative area of the double-ring complex of pro-Monalysin before and after trypsin injection. The average coverage area before trypsin injection is set to 1. The time course after injection is missing for 20–30 s. This is because the AFM images during this term were disturbed, and the area measurements cannot be performed.

We next visualized the conversion of pro-Monalysin to active-Monalysin after trypsin treatment in the PBS buffer. The video shows that, in a trypsin-concentration dependent manner, almost all molecules with a height of ~14 nm were converted into molecules with a height of ~5 nm over time (Figure 5, Supplementary Movie 4). Interestingly, we noticed that, by applying stronger tapping forces, the pro-form with a height of ~14 nm can be changed into molecules with a height of ~5 nm without trypsin treatment. Indeed, this change occurred depending on the strength of the tapping force (Supplementary Figures 7A–C, Supplementary Movie 5). When *A*_*sp*_*/A*_0_ were set at 0.5, this height change was seen for almost all the pro-form molecules after 60 s. The average tapping force in this imaging condition is estimated to be 53 ± 17 pN, using the nominal values of *A*_0_ = 2.0 ± 0.2 nm, *k*_*c*_ = 80 ± 20 pN/nm, and *Q*_*c*_ = 1.3 ± 0.2. In contrast, under the typical imaging conditions using *A*_*sp*_*/A*_0_ of more than 0.8, giving an average tapping force of <37 ± 17 pN, this height alteration was not seen at all, even after 60 s. These results strongly suggest that the pro-Monalysin height change seen in the trypsin treatment is induced by the proteolytic cleavage, not by the mechanical perturbations. Thus, the results seen in Figure 5 are direct evidence that trypsin treatment effectively digests a portion of pro-Monalysin and produces the active-forms by dissociating the doubly stacked disks. Note that the active-forms can withstand an average tapping force of ~50 pN (Supplementary Figures 7D,E, Supplementary Movie 6). In addition, the height alternation of the pro-form from 14 to 5 nm was also induced just when the molecules were strongly immobilized on the mica surface under the low salt buffer (Supplementary Figure 8, Supplementary Movie 7). These results suggest that the intramolecular interactions supporting the disk-shaped octamer structure are strong, while the disk-disk interaction is relatively weak and perhaps only sustained by the interaction of amino acid residues removed during protease activation. However, it remains an open question whether the force-indeed molecules from the pro-form with a height of ~5 nm are active and can form nanopores within the cell membrane. Importantly, the pro-Monalysin, not activated by trypsin treatment, strongly immobilized on the mica surface formed the disk-shaped octamer with the central pore (Supplementary Figure 8B). Consistent with the previous report (26), this result suggests that the central pore is already formed in the doubly stacked disks of the pro-Monalysin in solution.

### Real-Time Dynamics of Monalysin Insertion Into a Lipid Bilayer

**Figure 6:**
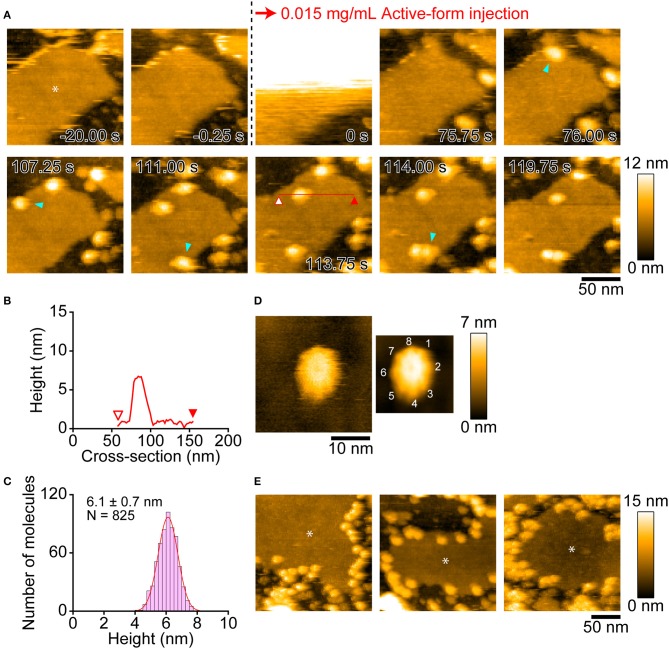
AFM images showing insertion of active-Monalysin onto lipid membrane formed on the PDMS surface. **(A)** Successive AFM images before and after active-Monalysin injection (Supplementary Movie 8). Dark area represents the PDMS surface, while slightly bright area represents the lipid membrane surface (see the asterisk in the first image). The scanning area was 150 × 150 nm^2^ with 80 × 80 pixels, and the imaging rate was 250 ms/frame. At 0 s, a drop of active-Monalysin solution was injected in the observation buffer. The final concentrations of active-Monalysin in the observation buffer were 0.015 mg/mL. After injection, the active-Monalysin were gradually inserted on the lipid bilayer (see the light blue arrowheads). **(B)** A cross-section analysis of active-Monalysin inserted in the lipid bilayer. The section is from the red line drawn on the image in **A**. **(C)** Height distributions of active-Monalysin inserted in the lipid bilayer. The distribution was fitted by single-Gaussian curve. **(D)** Small-area image of active-Monalysin inserted in the lipid bilayer (Supplementary Movie 9). The scanning area was 30 × 30 nm^2^ with 150 × 150 pixels, and the imaging rate was 330 ms/frame. The right image is an averaged image using 20 successive images. **(E)** AFM image gallery showing that the active-Monalysin are preferentially inserted into the edge of lipid bilayer. Asterisk marks represent the lipid bilayer areas. The scanning area was 200 × 200 nm^2^ with 100 × 100 pixels, and the imaging rate was 330 ms/frame.

We next visualized the insertion events of the active-Monalysin into a lipid membrane (Figure 6). A lipid membrane composed of a mixture of phospholipids of DOPC/DOPS/biotin-cap-DOPE was formed on the surface of PDMS (35). The active-Monalysin was then added into the observation buffer to be monitored. HS-AFM video showed that the active-Monalysin was inserted into the lipid membrane without significant structural change (Figure 6, Supplementary Movie 8). The active-form height was 6.1 ± 0.7 nm (mean ± S.D.) from the surface of lipid membrane (Figures 6B,C), which is slightly higher than that seen in the active-form on mica. At smaller scan sizes, the sub-unit stoichiometry was directly resolved to be 8-mer (Figure 6D, Supplementary Movie 9). These results collectively suggest that endogenous pro-Monalysin is a 16-mer complex, separated by protease into 8-mer active complexes, and the 8-mer active complex is inserted into the lipid membrane as they are.

Interestingly, Monalysin was preferentially inserted into the edge of the lipid membrane (Figure 6E). This implies that Monalysin prefers to be inserted in highly curved parts of the membrane. Consistent with this, we observed many insertions of active-Monalysin into the liposome (Supplementary Figure 9A, Supplementary Movie 10), while no insertion was seen into the lipid membrane formed on the mica surface (Supplementary Figure 9E). The surface roughness of the lipid bilayer formed on the PDMS and mica surfaces were 0.51 ± 0.08 nm and 0.13 ± 0.05 nm, respectively. These results indicate that the active-Monalysin can recognize such difference in the surface roughness of lipid membrane and make a drastic difference in the membrane insertion. Interestingly, the liposome placed on the mica surface was distorted and deformed into a disc shape (~13 nm in height and ~80 nm in width) rather than a spherical shape (Supplementary Figure 9B), and all Monalysin molecules were located on the outer periphery of the disc (see the blue arrowheads in Supplementary Figure 9A). This suggests that the active-form can spontaneously migrate to highly curved sites on the lipid membrane. In addition, we found that the active-Monalysin which have been reconstituted with liposome has two height states of 5.9 ± 0.5 and 7.3 ± 0.4 nm (Supplementary Figures 9C,D). Considering that the height of the active-form inserted into the lipid membrane formed on the PDMS is ~6 nm, the active molecule inserted into the curved membrane may have a height of ~7 nm, and the molecules with the height of ~7 nm seen in Supplementary Figures 9C,D are presumably in a molecular state before height transition from ~7 to ~6 nm.

## Discussion

In this study, we reported, for the first time, on the purification of endogenous pro-Monalysin from entomopathogenic bacteria *P. entomophila*. Purified pro-Monalysin is activated by trypsin treatment, which is confirmed by electrophysiological analysis with an artificial lipid membrane. The pro-form is stable for more than a month at 4°C (data not shown), and active-form produced by trypsin treatment had cytotoxic activity in *Drosophila* cell line and adult flies. In particular, we examined the distinct structure and dynamics of endogenous Monalysin in solution and within the lipid membrane using HS-AFM and revealed the stability of the active-octamer structure. This study suggests that endogenous Monalysin is one of the best model toxins from entomopathogenic bacteria. Additionally, information on pore size estimated by electrophysiological analysis is useful for the potential development of biological nanopores from endogenous Monalysin.

We purified pro-Monalysin based on cytotoxic activity from cell pellets of *P. entomophila*, not from a culture supernatant, meaning that a large amount of Monalysin is kept inside the cells as an assembled pro-form. The diameters of pro-Monalysin and active-Monalysin, estimated from HS-AFM analysis, are ~11 nm and their heights are ~14 and 5–7 nm, respectively. Both sizes are too large to be secreted by *P. entomophila's* secretion system. It has type I and II secretion systems with secretion pore diameters that are generally <5 nm (48–50). Since *P. entomophila* tends to undergo autolysis, particularly at temperatures over 30°C, some *P. entomophila* may have been lysed in the host's intestine. In this case, pro-Monalysin was released from the dead bacteria and digested by bacterial proteases such as AprA, or perhaps also by host proteases, to become its active-form. We also observed that the trefoil-shaped structure of 16-mer pro-Monalysin composes the trimer complex in the PBS buffer when the concentration of pro-Monalysin is high enough. By making a trimer complex, the cleavage site for proteolytic activation of pro-Monalysin might be hidden and prevented from being an active-form in *P. entomophila*. Sub-cellular localization and estimated concentration of pro-Monalysin in *P. entomophila* should be investigated in a future study.

The most well-characterized PFTs that damage insect tissue are probably Cry toxins from *Bacillus thuringiensis*, a Gram-positive bacterium commonly used as a biological pesticide (27). Cry toxins represent a large family, consisting of more than 350 different members, yet their common features as toxins are essentially the same (27). They form crystalline inclusions after production, are solubilized, undergo partial cleavage by proteases in digestive juice, and are activated after ingestion. The activated Cry toxin is considered a monomer and then forms a pore on the cell membrane of midgut epithelial cells, following specific interactions with a receptor(s), resulting in cell lysis and destruction of midgut tissue (27, 28). However, our study showed that endogenous Monalysin was composed of a pore-forming multimer from the beginning and demonstrates a receptor-independent insertion into the lipid membrane. Importantly, since Cry toxins show target specificity of insect species through selective toxin-receptor interactions, one needs to employ a genetic trick when using them on flies, e.g., overexpression of the Cry1Aa receptor and the application of Cry1Aa thereafter (29). Thus, if one seeks to impose tissue damage in a non-specific manner, Monalysin injection or ingestion would be a simple method. Indeed, our study showed that injection of endogenous Monalysin, through a standard procedure, effectively killed adult flies and induced an innate immune response. This study provides a theoretical basis for the use of endogenous Monalysin toxins as a tool for studying injury-induced innate immunity in the context of microbial infections. Note that endogenous Monalysin can be purified milligram order from 1 L of bacterial culture, suggesting its versatility within a range of experiments.

**Figure 7:**
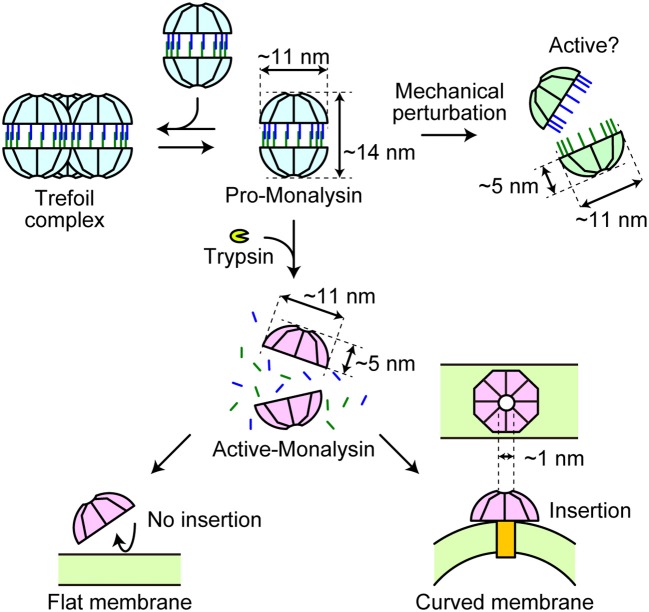
A model of Monalysin activation for pore formation. Endogenous pro-Monalysin presents a 16-mer complex and occasionally forms a trefoil-shaped structure composed of trimer complex in the PBS buffer when its concentration is high enough. After trypsin treatment or mechanical perturbation, the double-stacked disk-like 16-mer complex dissociates into two disk-shaped 8-mer complexes. The 8-mer complex of active-Monalysin, in turn, preferentially inserts itself into the curved lipid membrane and forms nanopores (pore size = 1 nm).

Our HS-AFM analysis revealed real-time dynamics of Monalysin in action (Figure 7). Pro-Monalysin purified from *P. entomophila* showed a relatively scissile 16-mer complex (though stable enough during the purification step and storage), in contrast to the 18-mer structure of recombinant Monalysin prepared from *E. coli*. A 16-mer model of our endogenous Monalysin is consistent with gel filtration chromatography and MALDI-TOFMS analysis. This discrepancy might be derived from the different techniques, or the hosts, used to express Monalysin. Alternatively, pore-forming toxins tend to compose different subunit stoichiometry in solution than in crystal. Indeed, α-hemolysin had been suggested to form heptamer by X-ray crystallography, though an AFM analysis indicated that α-hemolysin composes hexameric stoichiometry (51). This report and our current study may imply that pore-forming toxins, in general, could form two different energetically stable oligomers in different conditions. By using endogenous Monalysin, it would be interesting to solve crystal structure or to perform cryo-EM in the future study.

Activated-Monalysin obtained by trypsin treatment has an 8-mer constitution and roughly halves in height, indicating that pro-Monalysin is half dissociated into an 8-mer pair well before insertion. It penetrates and forms pores in the lipid bilayer without profound structural change. Notably, Monalysin was preferentially inserted into the edge of the lipid membrane, implying that Monalysin could recognize the target membrane's curvature. Eukaryotic cells possess local membrane subdomains, some of which have high curved areas, such as the tip of filopodia and the area of endocytosis or exocytosis. Those subregions have important biological functions for cell movement, intracellular communication, and signaling (52). Phagocytosing immune cells, such as macrophages, extend a lot of filopodia, particularly upon immune activation. Monalysin or its relatives might preferentially target those cells and/or important biological membrane regions. Additionally, a possible entry site of Monalysin is the tip of microvilli of enterocytes in the fly's intestinal epithelium, as previous studies have reported that Monalysin killed adult flies after oral infection through gut injury (23). Furthermore, electrophysiological analysis revealed that Monalysin inserts itself most commonly within lipid bilayers with high ratio of PC. The chemical nature of lipids determines how the lipids bundle side-by-side in a monolayer and thereby influences the monolayer curvature. For example, lysophospholipids form positively curved monolayers; PC build nearly flat monolayers; and DOPE assemble negatively curved monolayers (53). Notably, upon cell activation, some of the membrane phospholipids are metabolized into eicosanoids and lysophospholipids (54). Active-Monalysin may prefer to insert a highly bent portion of the plasma membrane with a large amount of lysophospholipids produced upon a cell signaling event. From this point of view, not only local membrane subdomains, but also some specific cell types and/or cell activation status could be the insertion target of active-Monalysin. And then, these characteristics might determine host and position specificity where Monalysin shows toxicity. More detailed analysis to find more specific lipid preference and the optimal radius of curvature for Monalysin insertion shall be conducted *in vitro* and *in vivo*. Further studies on endogenous Monalysin are required to answer questions concerning the precise mode-of-action during pore formation in the target membrane.

## Data Availability Statement

All datasets generated for this study are included in the article/Supplementary Material.

## Author Contributions

NK and TK: conceptualization. SN, KK, NK, and TK: formal analysis and writing—original draft. SN, ES, KK, AH, FN, RA, AM, TN, ST, NK, and TK: investigation. SN, ES, KK, AH, ST, NK, and TK: funding acquisition. All authors writing—review and editing.

### Conflict of Interest

The authors declare that the research was conducted in the absence of any commercial or financial relationships that could be construed as a potential conflict of interest.

## References

[1] Akira S Uematsu S Takeuchi O. Pathogen recognition and innate immunity. Cell. (2006) 124:783–801. 10.1016/j.cell.2006.02.015 16497588

[2] Chen GY Nunez G. Sterile inflammation: sensing and reacting to damage. Nat Rev Immunol. (2010) 10:826–37. 10.1038/nri2873 21088683 PMC3114424

[3] Venereau E Ceriotti C Bianchi ME. DAMPs from cell death to new life. Front Immunol. (2015) 6:422. 10.3389/fimmu.2015.00422 26347745 PMC4539554

[4] Lemaitre B Hoffmann J. The host defense of *Drosophila* melanogaster. Annu Rev Immunol. (2007) 25:697–743. 10.1146/annurev.immunol.25.022106.141615 17201680

[5] Myllymaki H Valanne S Ramet M. The *Drosophila* imd signaling pathway. J Immunol. (2014) 192:3455–62. 10.4049/jimmunol.1303309 24706930

[6] Valanne S Wang JH Ramet M. The *Drosophila* toll signaling pathway. J Immunol. (2011) 186:649–56. 10.4049/jimmunol.1002302 21209287

[7] Gobert V Gottar M Matskevich AA Rutschmann S Royet J Belvin M. Dual activation of the *Drosophila* toll pathway by two pattern recognition receptors. Science. (2003) 302:2126–30. 10.1126/science.1085432 14684822

[8] Michel T Reichhart JM Hoffmann JA Royet J. *Drosophila* toll is activated by gram-positive bacteria through a circulating peptidoglycan recognition protein. Nature. (2001) 414:756–9. 10.1038/414756a 11742401

[9] Gottar M Gobert V Matskevich AA Reichhart JM Wang C Butt TM. Dual detection of fungal infections in *Drosophila* via recognition of glucans and sensing of virulence factors. Cell. (2006) 127:1425–37. 10.1016/j.cell.2006.10.046 17190605 PMC1865096

[10] Parthier C Stelter M Ursel C Fandrich U Lilie H Breithaupt C. Structure of the Toll-Spatzle complex, a molecular hub in *Drosophila* development and innate immunity. Proc Natl Acad Sci USA. (2014) 111:6281–6. 10.1073/pnas.1320678111 24733933 PMC4036000

[11] Sun H Bristow BN Qu G Wasserman SA. A heterotrimeric death domain complex in toll signaling. Proc Natl Acad Sci USA. (2002) 99:12871–6. 10.1073/pnas.202396399 12351681 PMC130552

[12] Lindsay SA Wasserman SA. Conventional and non-conventional *Drosophila* toll signaling. Dev Comp Immunol. (2014) 42:16–24. 10.1016/j.dci.2013.04.011 23632253 PMC3787077

[13] Kleino A Silverman N. The *Drosophila* IMD pathway in the activation of the humoral immune response. Dev Comp Immunol. (2014) 42:25–35. 10.1016/j.dci.2013.05.014 23721820 PMC3808521

[14] Paquette N Broemer M Aggarwal K Chen L Husson M Erturk-Hasdemir D. Caspase-mediated cleavage, IAP binding, and ubiquitination: linking three mechanisms crucial for *Drosophila* NF-kappaB signaling. Mol Cell. (2010) 37:172–82. 10.1016/j.molcel.2009.12.036 20122400 PMC2819219

[15] El Chamy L Leclerc V Caldelari I Reichhart JM. Sensing of danger signals and pathogen-associated molecular patterns defines binary signaling pathways upstream of Toll. Nat Immunol. (2008) 9:1165–70. 10.1038/ni.1643 18724373 PMC2768518

[16] Ming M Obata F Kuranaga E Miura M. Persephone/spatzle pathogen sensors mediate the activation of toll receptor signaling in response to endogenous danger signals in apoptosis-deficient *Drosophila*. J Biol Chem. (2014) 289:7558–68. 10.1074/jbc.M113.543884 24492611 PMC3953269

[17] Issa N Guillaumot N Lauret E Matt N Schaeffer-Reiss C Van Dorsselaer A. The circulating protease persephone is an immune sensor for microbial proteolytic activities upstream of the *Drosophila* toll pathway. Mol Cell. (2018) 69:539–50. 10.1016/j.molcel.2018.01.029 29452635 PMC5823974

[18] Arefin B Kucerova L Dobes P Markus R Strnad H Wang Z. Genome-wide transcriptional analysis of *Drosophila* larvae infected by entomopathogenic nematodes shows involvement of complement, recognition and extracellular matrix proteins. J Innate Immun. (2014) 6:192–204. 10.1159/000353734 23988573 PMC6741570

[19] Hyrsl P Dobes P Wang Z Hauling T Wilhelmsson C Theopold U. Clotting factors and eicosanoids protect against nematode infections. J Innate Immun. (2011) 3:65–70. 10.1159/000320634 20948189

[20] Vodovar N Vinals M Liehl P Basset A Degrouard J Spellman P. *Drosophila* host defense after oral infection by an entomopathogenic pseudomonas species. Proc Natl Acad Sci USA. (2005) 102:11414–9. 10.1073/pnas.0502240102 16061818 PMC1183552

[21] Martins NE Faria VG Teixeira L Magalhaes S Sucena E. Host adaptation is contingent upon the infection route taken by pathogens. PLoS Pathog. (2013) 9:e1003601. 10.1371/journal.ppat.1003601 24086131 PMC3784483

[22] Chakrabarti S Liehl P Buchon N Lemaitre B. Infection-induced host translational blockage inhibits immune responses and epithelial renewal in the *Drosophila* gut. Cell Host Microbe. (2012) 12:60–70. 10.1016/j.chom.2012.06.001 22817988

[23] Opota O Vallet-Gely I Vincentelli R Kellenberger C Iacovache I Gonzalez MR. Monalysin, a novel β-pore-forming toxin from the *Drosophila* pathogen *Pseudomonas entomophila*, contributes to host intestinal damage and lethality. PLoS Pathog. (2011) 7:e1002259. 10.1371/journal.ppat.1002259 21980286 PMC3182943

[24] Kuraishi T Binggeli O Opota O Buchon N Lemaitre B. Genetic evidence for a protective role of the peritrophic matrix against intestinal bacterial infection in *Drosophila* melanogaster. Proc Natl Acad Sci USA. (2011) 108:15966–71. 10.1073/pnas.1105994108 21896728 PMC3179054

[25] Shibata T Maki K Hadano J Fujikawa T Kitazaki K Koshiba T. Crosslinking of a peritrophic matrix protein protects gut epithelia from bacterial exotoxins. PLoS Pathog. (2015) 11:e1005244. 10.1371/journal.ppat.1005244 26506243 PMC4646701

[26] Leone P Bebeacua C Opota O Kellenberger C Klaholz B Orlov I. X-ray and cryo-electron microscopy structures of monalysin pore-forming toxin reveal multimerization of the pro-form. J Biol Chem. (2015) 290:13191–201. 10.1074/jbc.M115.646109 25847242 PMC4505573

[27] Jurat-Fuentes JL Crickmore N. Specificity determinants for cry insecticidal proteins: insights from their mode of action. J Invertebr Pathol. (2017) 142:5–10. 10.1016/j.jip.2016.07.018 27480404

[28] Bravo A Gómez I Porta H García-Gómez BI Rodriguez-Almazan C Pardo L. Evolution of *Bacillus thuringiensis* cry toxins insecticidal activity. Microb Biotechnol. (2013) 6:17–26. 10.1111/j.1751-7915.2012.00342.x 22463726 PMC3815381

[29] Obata F Tanaka S Kashio S Tsujimura H Sato R Miura M. Induction of rapid and selective cell necrosis in *Drosophila* using *Bacillus thuringiensis* cry toxin and its silkworm receptor. BMC Biol. (2015) 13:48. 10.1186/s12915-015-0160-2 26152191 PMC4495774

[30] Jetha NN Wiggin M Marziali A. Forming an alpha-hemolysin nanopore for single-molecule analysis. Methods Mol Biol. (2009) 544:113–27. 10.1007/978-1-59745-483-4_9 19488697

[31] Asano T Nishiuchi T. Comparative analysis of phosphoprotein expression using 2D-DIGE. Methods Mol Biol. (2011) 744:225–33. 10.1007/978-1-61779-123-9_16 21533697

[32] Kenmoku H Ishikawa H Ote M Kuraishi T Kurata S. A subset of neurons controls the permeability of the peritrophic matrix and midgut structure in *Drosophila* adults. J Exp Biol. (2016) 219:2331–9. 10.1242/jeb.122960 27229474

[33] Ando T Uchihashi T Fukuma T High-speed atomic force microscopy for nano-visualization of dynamic biomolecular processes. Prog Surf Sci. (2008) 83:337–437. 10.1016/j.progsurf.2008.09.001

[34] Uchihashi T Kodera N Ando T. Guide to video recording of structure dynamics and dynamic processes of proteins by high-speed atomic force microscopy. Nat Protoc. (2012) 7:1193–206. 10.1038/nprot.2012.047 22635111

[35] Uchihashi T Watanabe H Kodera N. Optimum substrates for imaging biological molecules with high-speed atomic force microscopy. Methods Mol Biol. (2018) 1814:159–79. 10.1007/978-1-4939-8591-3_10 29956232

[36] Ngo KX Kodera N Katayama E Ando T Uyeda TQ. Cofilin-induced unidirectional cooperative conformational changes in actin filaments revealed by high-speed atomic force microscopy. Elife. (2015) 4:e04806. 10.7554/eLife.04806.033 25642645 PMC4337605

[37] Manaka J Kuraishi T Shiratsuchi A Nakai Y Higashida H Henson P. Draper-mediated and phosphatidylserine-independent phagocytosis of apoptotic cells by *Drosophila* hemocytes/macrophages. J Biol Chem. (2004) 279:48466–76. 10.1074/jbc.M408597200 15342648

[38] Obata F Tsuda-Sakurai K Yamazaki T Nishio R Nishimura K Kimura M. Nutritional control of stem cell division through S-adenosylmethionine in *Drosophila* intestine. Dev Cell. (2018) 44:741–51. 10.1016/j.devcel.2018.02.017 29587144

[39] Buchon N Broderick NA Kuraishi T Lemaitre B. *Drosophila* EGFR pathway coordinates stem cell proliferation and gut remodeling following infection. BMC Biol. (2010) 8:152. 10.1186/1741-7007-8-152 21176204 PMC3022776

[40] Funakoshi K Suzuki H Takeuchi S. Lipid bilayer formation by contacting monolayers in a microfluidic device for membrane protein analysis. Anal Chem. (2006) 78:8169–74. 10.1021/ac0613479 17165804

[41] Gutsmann T Heimburg T Keyser U Mahendran KR Winterhalter M. Protein reconstitution into freestanding planar lipid membranes for electrophysiological characterization. Nat Protoc. (2015) 10:188–98. 10.1038/nprot.2015.003 25551663

[42] Ando T Uchihashi T Scheuring S. Filming biomolecular processes by high-speed atomic force microscopy. Chem Rev. (2014) 114:3120–88. 10.1021/cr4003837 24476364 PMC4076042

[43] Ando T Kodera N Takai E Maruyama D Saito K Toda A. A high-speed atomic force microscope for studying biological macromolecules. Proc Natl Acad Sci USA. (2001) 98:12468–72. 10.1073/pnas.211400898 11592975 PMC60077

[44] Czajkowsky DM Hotze EM Shao Z Tweten RK. Vertical collapse of a cytolysin prepore moves its transmembrane beta-hairpins to the membrane. EMBO J. (2004) 23:3206–15. 10.1038/sj.emboj.7600350 15297878 PMC514522

[45] Yilmaz N Yamada T Greimel P Uchihashi T Ando T Kobayashi T. Real-time visualization of assembling of a sphingomyelin-specific toxin on planar lipid membranes. Biophys J. (2013) 105:1397–405. 10.1016/j.bpj.2013.07.052 24047991 PMC3785888

[46] Leung C Dudkina NV Lukoyanova N Hodel AW Farabella I Pandurangan AP. Stepwise visualization of membrane pore formation by suilysin, a bacterial cholesterol-dependent cytolysin. Elife. (2014) 3:e04247. 10.7554/eLife.04247.021 25457051 PMC4381977

[47] Ni T Jiao F Yu X Aden S Ginger L Williams SI. Structure and mechanism of bactericidal mammalian perforin-2, an ancient agent of innate immunity. Sci Adv. (2020) 6:eaax8286. 10.1126/sciadv.aax8286 32064340 PMC6989145

[48] Vodovar N Vallenet D Cruveiller S Rouy Z Barbe V Acosta C. Complete genome sequence of the entomopathogenic and metabolically versatile soil bacterium *Pseudomonas entomophila*. Nat Biotechnol. (2006) 24:673–9. 10.1038/nbt1212 16699499

[49] Kim JS Song S Lee M Lee S Lee K Ha NC. Crystal structure of a soluble fragment of the membrane fusion protein HlyD in a type I secretion system of gram-negative bacteria. Structure. (2016) 24:477–85. 10.1016/j.str.2015.12.012 26833388

[50] Hay ID Belousoff MJ Lithgow T. Structural basis of type 2 secretion system engagement between the inner and outer bacterial membranes. mBio. (2017) 8:e01344–17. 10.1128/mBio.01344-17 29042496 PMC5646249

[51] Czajkowsky DM Sheng S Shao Z. Staphylococcal alpha-hemolysin can form hexamers in phospholipid bilayers. J Mol Biol. (1998) 276:325–30. 10.1006/jmbi.1997.1535 9512705

[52] McMahon HT Boucrot E. Membrane curvature at a glance. J Cell Sci. (2015) 128:1065–70. 10.1242/jcs.114454 25774051 PMC4359918

[53] Graham TR Kozlov MM. Interplay of proteins and lipids in generating membrane curvature. Curr Opin Cell Biol. (2010) 22:430–6. 10.1016/j.ceb.2010.05.002 20605711 PMC3770468

[54] Hla T Lee MJ Ancellin N Paik JH Kluk MJ. Lysophospholipids–receptor revelations. Science. (2001) 294:1875–8. 10.1126/science.1065323 11729304

