# Supplementary material for: Molecular and Functional Analysis of Pore-Forming Toxin Monalysin From Entomopathogenic Bacterium Pseudomonas entomophila
Source: Front Immunol. 2020 Mar 27;11:520. doi: 10.3389/fimmu.2020.00520 (PMC7118224; doi:10.3389/fimmu.2020.00520)
Supplement: Supplementary file 11 [file Data_Sheet_1.docx]

Supplementary Material

# Supplementary Figures and Tables


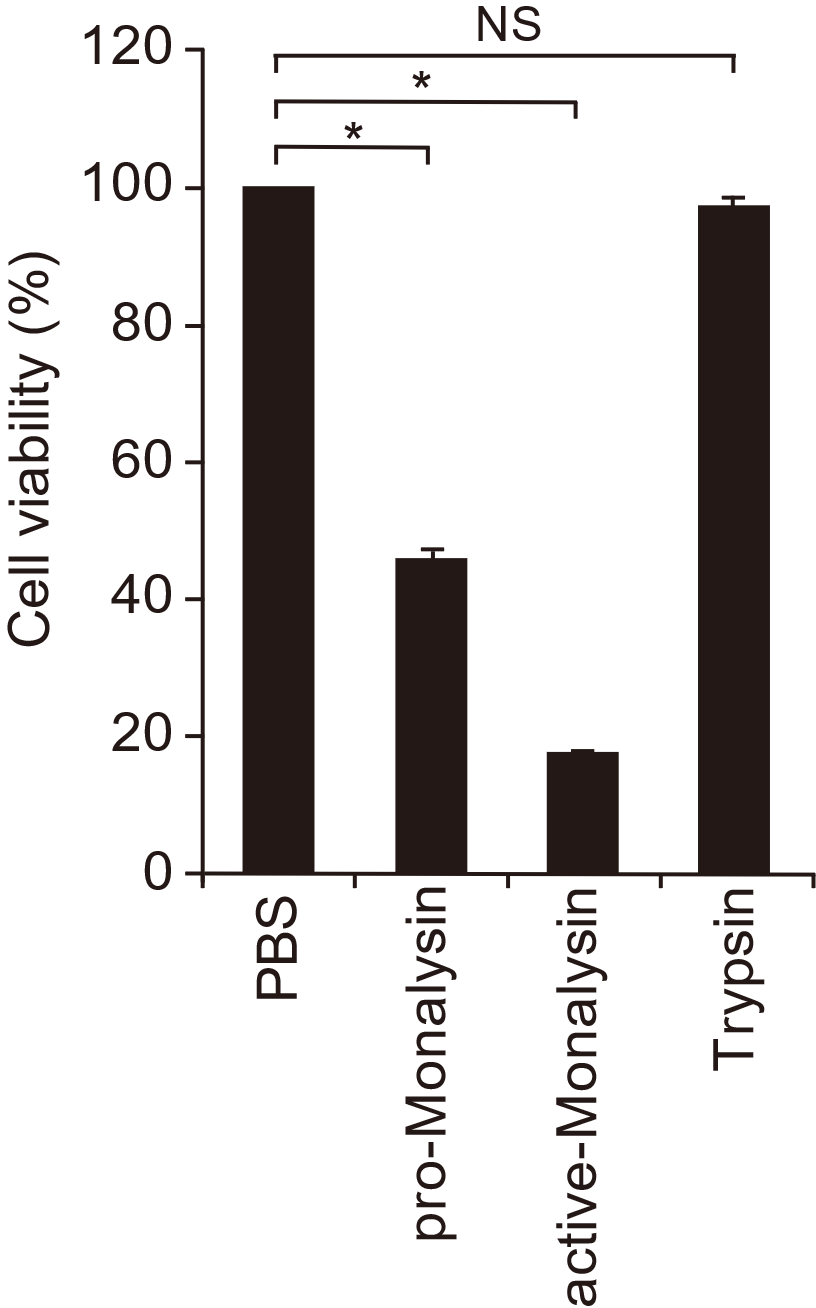


**Supplementary Figure 1**

Cell viability after incubation with pro-Monalysin, active-Monalysin and Trypsin. Cell viability is shown relative to luminescence in cells incubated with PBS, taken as 100 %. The means ± S.E. obtained with the data from triplicate samples in two independent experiments are presented (**P* < 0.05; NS: not significant, as determined by the Student’s *t*-test).


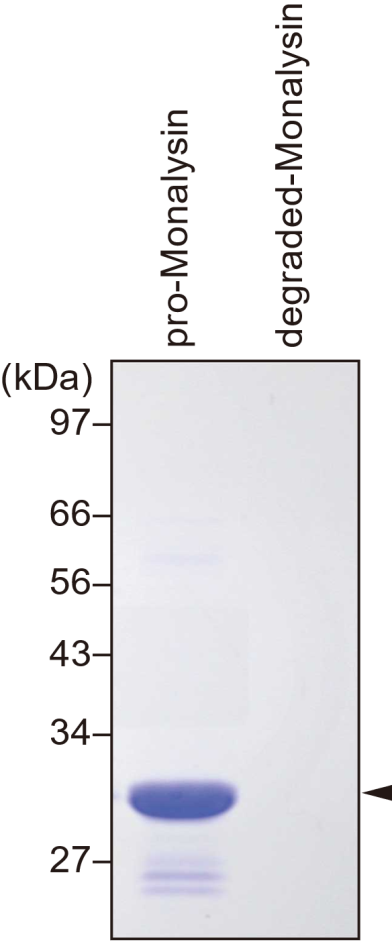


**Supplementary Figure 2**

A SDS-PAGE analysis of purified pro-Monalysin and degraded-Monalysin. To degrade Monalysin completely, trypsin was added to pro-Monalysin (10 μg) at 0.2 mg/mL and incubated at 37°C for 58 h. 10 μg of pro-Monalysin and degraded Monalysin were loaded in 12% (w/v) SDS-acrylamide gel and stained with Coomassie Brilliant Blue. The arrowhead indicates pro-Monalysin.

**Supplementary Figure 3**

**(A)** Quantification of PH3-positive cells per gut in *dcy^1^* flies at 8 h after oral ingestion of 2 mg/mL of active-Monalysin. (−) indicates sucrose feeding and (+) indicates active-Monalysin feeding. In total, 18 and 32 guts were observed in flies of (−) or (+) oral ingestion, respectively. **(B)** The confocal microscopy images of posterior midgut in *dcy^1^* flies without (−) or with (+) oral injection of active-Monalysin. The septate junction marker Dlg (green) and nuclei (blue) were visualized using anti-Dlg antibodies and DAPI. Scale bar; 10 μm.


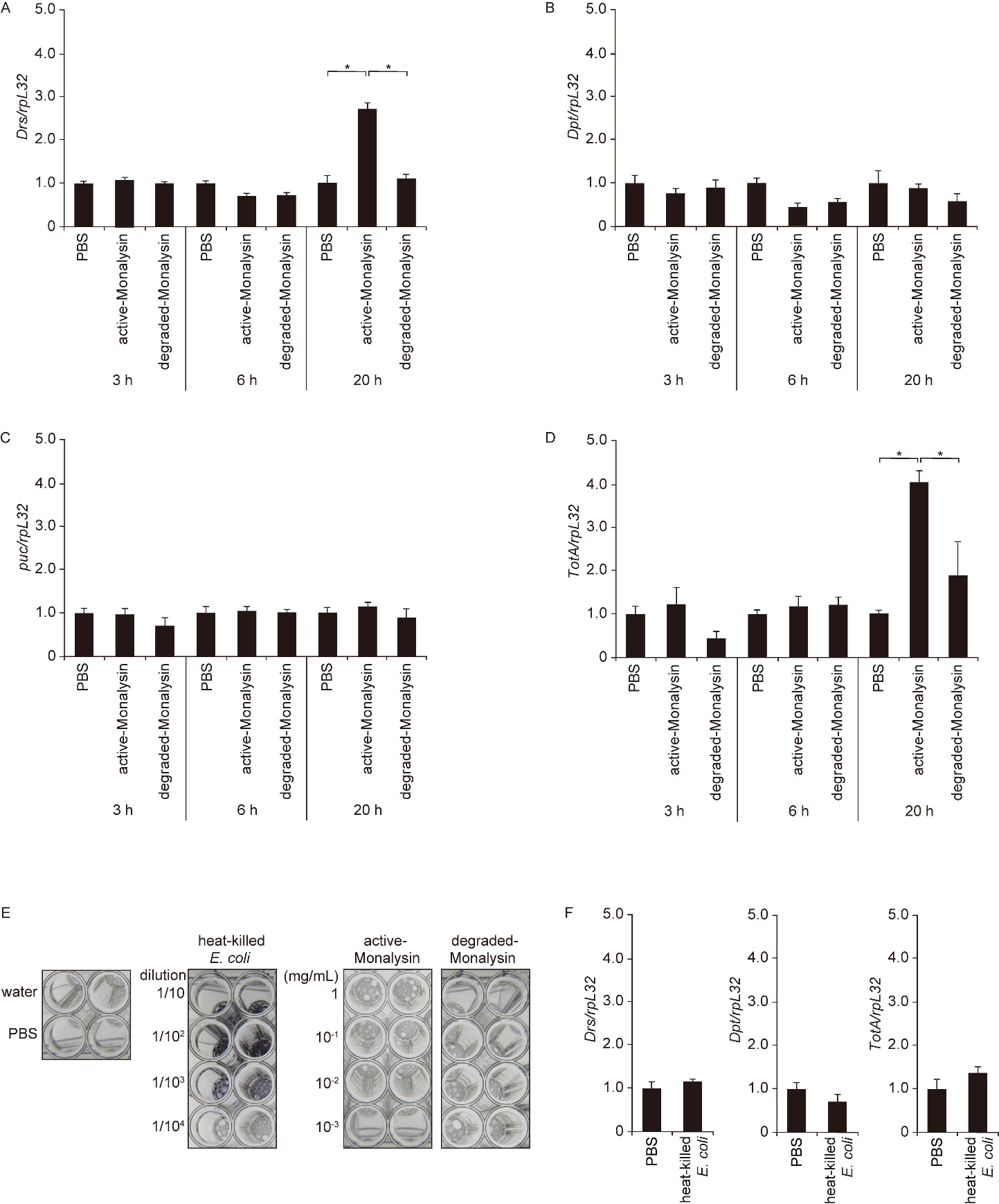


**Supplementary Figure 4**

Systemic injection of Monalysin inducing humoral immunity and stress response in adult flies. **(A-D)** Real-time qPCR analysis of *Drosomycin* (*Drs*) (A), *Diptericin* (*Dpt*) (B), *puckered* (*puc*) (C)*,* *Turandot A* (*TotA*) (D) expression in adult flies at 3, 6, 20 h after injection of PBS, 50 μg/mL active-Monalysin, or 50 μg/mL degraded-Monalysin; *rpL32* was used as an internal control. The data is shown, relative to the value in PBS-injected flies at each time point, taken as 1.0. The means ± S.E. obtained with the data from 4-6 samples (one sample is derived from 10 flies) are presented. (**P* < 0.0001, determined by the Student’s *t*-test). **(E)** Contamination level of peptidoglycan in purified Monalysin. Contamination level were monitored as blackness derived from melanin pigment which are produced in sample containing peptidoglycan. As a positive control, heat-killed *E. coli* were subjected to the same test. **(F)** Real-time qPCR analysis of *Drs* (left), *Dpt* (middle) and *TotA* (right) expression in adult flies at 20 h after injection of PBS and 1000 times dilution (1/10^3^) of heat-killed *E. coli*. The data is shown, relative to the value in PBS-injected flies at each time point, taken as 1.0. The means ± S.E. obtained with the data from seven samples are presented in all experiments.

**
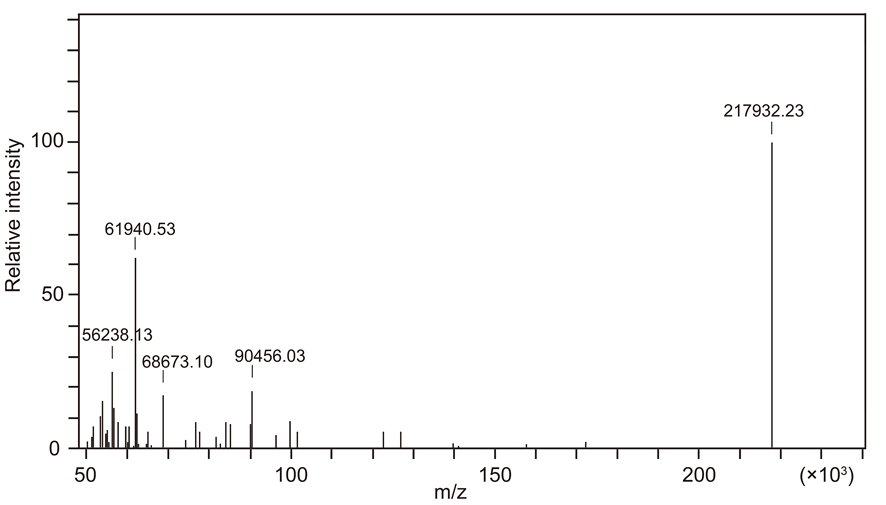
**

**Supplementary Figure 5**

Active-Monalysin solution, which had been obtained from the short trypsin treatment of pro-Monalysin, was subjected to an MALDI-TOF/TOF analysis. The mass spectrum shows integrated data via analysis software. The highest signal, whose m/z was 217932.23, indicates a possible molecular ion peak of active-Monalysin multimers.

**
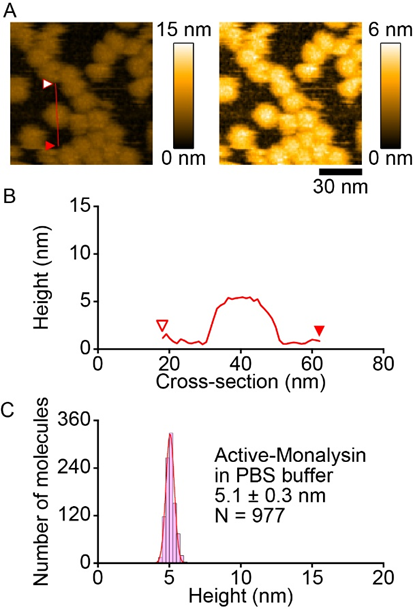
**

**Supplementary Figure 6**

Molecular features of active-Monalysin on mica surface in PBS buffer. **(A)** AFM images of active-Monalysin in a PBS buffer (See **Supplementary Movie 2**). Two different height scale images are shown. The scanning area was 100 × 100 nm^2^ with 100 × 100 pixels and the imaging rate was 250 ms/frame. **(B)** Cross-section analysis. The section is from the red line drawn on the images in **A**. **(C)** Height distributions of active-Monalysin observed in PBS buffer. The distribution was fitted by a single-Gaussian curve.


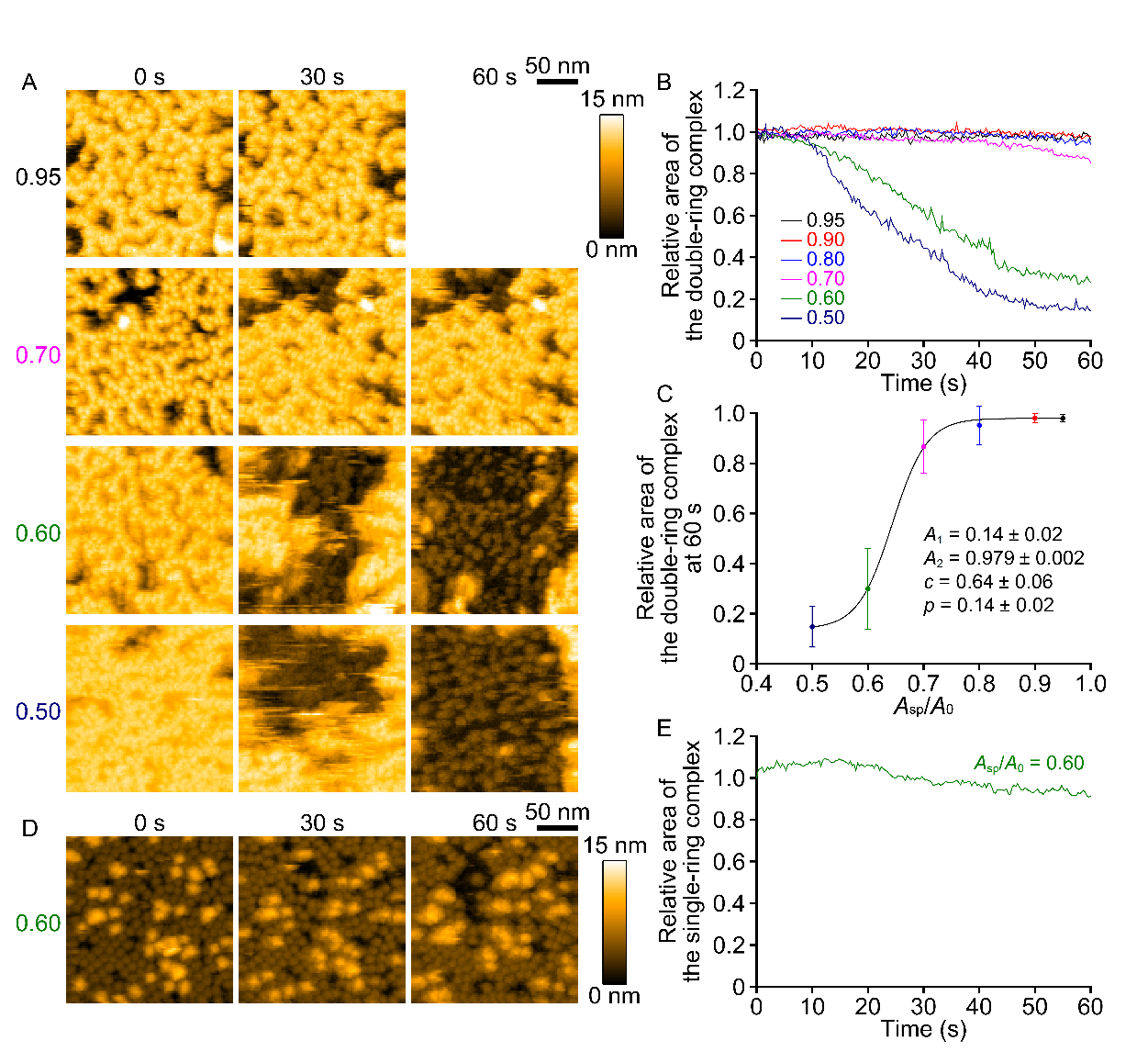


**Supplementary Figure 7**

AFM imaging of Monalysin under different tapping forces. **(A)** AFM images of pro-Monalysin under the different *A_sp_/A_0_* conditions (Supplementary Movie 5). From the top, *A_sp_/A_0_* was set to 0.95, 0.70, 0.60 and 0.50, respectively. The scanning area was 200 × 200 nm^2^ with 100 × 100 pixels and the imaging rate was 330 ms/frame. **(B)** The time course of the relative area of double-ring complex of pro-Monalysin after setting of *A_sp_/A_0_.* The line colors correspond to the settings of *A_sp_/A_0_*. The coverage area of the first image is set to 1. Each time course is an average one calculated from more than three different measurements. Note that the area of double-ring complex of pro-Monalysin was calculated by counting the pixels whose height was more than 8 nm. **(C)** The relative area of the double-ring complex at 60 s as a function of *A_sp_/A_0_*. Each data point was calculated from the same measurements described above. The data points were fitted by the dose-response relationship curve as follows.

$$y=A_{1}+\frac{A_{2}-A_{1}}{1+{10}^{p(c-x)}}$$

The fitting parameters are shown in the graph. **(D)** AFM image of active-Monalysin under the *A_sp_/A_0_* of 0.6 (**Supplementary Movie 6**). The scanning area was 200 × 200 nm^2^ with 100 × 100 pixels, and the imaging rate was 330 ms/frame. **(E)** The time course of the relative area of the single-ring complex of active-Monalysin after setting of *A_sp_/A_0_*. The coverage area of the first image is set to 1. Note that the area of the single-ring complex was calculated by counting the pixels whose height was in a range from 4 to 8 nm, meaning that the measurement errors from the adsorbed debris were minimized.

**
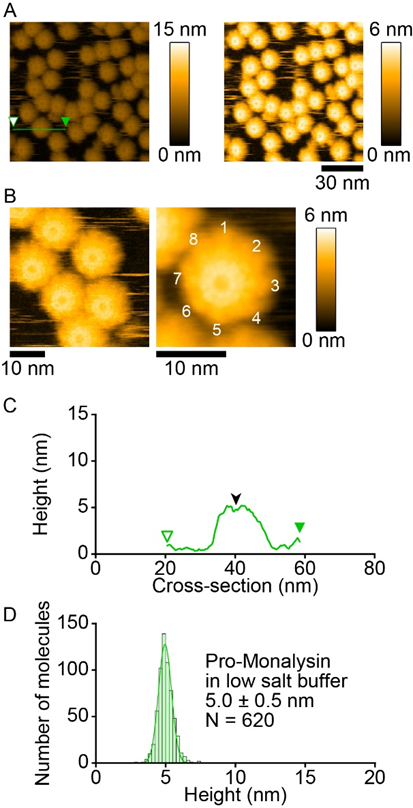
**

**Supplementary Figure 8**

Molecular features of pro-Monalysin on mica surface in a low salt buffer (30 mM NaCl, 10 mM Sodium phosphate, pH7.0). **(A)** A wide-area image of pro-Monalysin in the low salt buffer. Images of two different height scales are shown. The scanning area was 100 × 100 nm^2^ with 300 × 300 pixels, and the imaging rate was 1 s/frame. **(B)** Small-area image of pro-Monalysin in the low salt buffer (See **Supplementary Movie 7**). The scanning area was 40 × 40 nm^2^ with 200 × 200 pixels, and the imaging rate was 0.5 s/frame. **(C)** Cross-section analysis. The section is from the green line drawn on the images in A. The black arrowhead indicates the central pore. **(D)** Height distributions of pro-Monalysin observed in the low salt buffer. The distribution was fitted by a single-Gaussian curve.

**
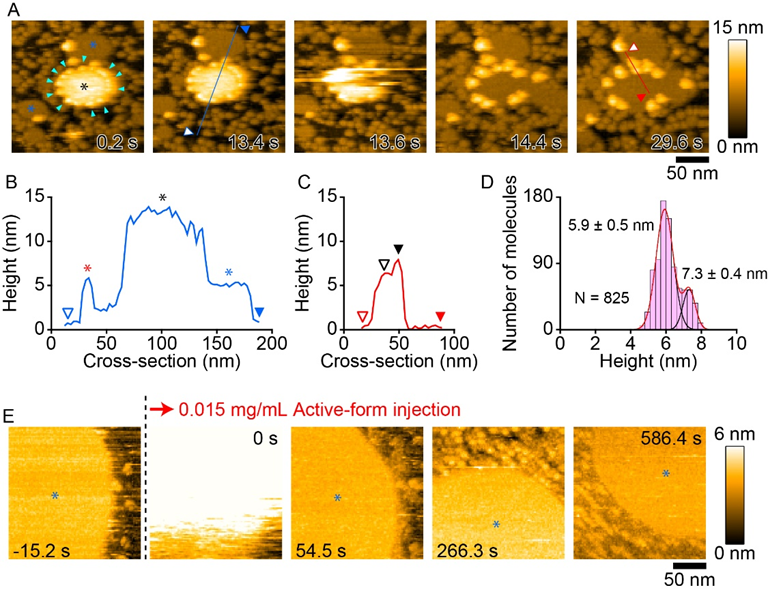
**

**Supplementary Figure 9**

Active-Monalysin are inserted into the curved membrane preferentially but not into the flat membrane. **(A)** Successive AFM images showing rupture of a liposome on which the active-Monalysin are inserted (See **Supplementary Movie 10**). Black and blue asterisks shown in the first image represents the surfaces of liposome and the lipid bilayer form on the mica surface. Light blue arrowheads in the first image represent the active-Monalysin molecules inserted in the liposome. At 13.6 s, the liposome ruptured into a flat membrane, after which the active-Monalysin pre-inserted into the lipid membrane were slowly diffusing on the membrane. The scanning area was 200 × 200 nm^2^ with 80 × 80 pixels, and the imaging rate was 200 ms/frame. **(B, C)** A cross-section analysis. The sections are from the blue and red lines drawn on the images in **A**. Black, blue and red asterisks in **B** represent the positions of liposome, lipid bilayer surface and active-Monalysin on mica surface. Black open and closed arrowheads in **C** represent the positions of the ~6 nm and ~7 nm spices of active-Monalysin, respectively. **(D)** Height distribution of active-Monalysin inserted on the lipid bilayer which is formed on mica surface by pre-incubated liposome with the active-Monalysin. The distribution was fitted by double-Gaussian curve. **(E)** Successive AFM images showing that the active-Monalysin are not inserted into the flat lipid bilayer formed on the mica surface. Blue asterisks represent the surfaces of lipid bilayer form on the mica surface. At 0 s, a drop of active-Monalysin solution was injected in the observation buffer. The final concentrations of active-Monalysin in the observation buffer were 0.015 mg/mL. Even after 580 s, no active-Monalysin was inserted into the lipid bilayer. The scanning area was 200 × 200 nm^2^ with 100 × 100 pixels, and the imaging rate was 330 ms/frame.

**Supplementary Movie legend**

**Supplementary Movie 1**

Pro-Monalysin on mica surface in the PBS buffer. The scanning area was 100 × 100 nm^2^ with 100 × 100 pixels and the imaging rate was 250 ms/frame. The height scale is 0 – 15 nm. The movie is played at 4 frames/s. Light blue arrowheads represent the dissociation and re-binding events of the pro-Monalysin.

**Supplementary Movie 2**

Active-Monalysin on mica surface in the PBS buffer. The scanning area was 100 × 100 nm^2^ with 100 × 100 pixels and the imaging rate was 250 ms/frame. The height scales of the right and left movies are 0 – 15 nm and 0 – 6 nm, respectively. The movie is played at 4 frames/s.

**Supplementary Movie 3**

Active-Monalysin on mica surface in the low salt buffer. The scanning area was 40 × 40 nm^2^ with 120 × 120 pixels and the imaging rate was 150 ms/frame. The height scales of the right and left movies are 0 – 15 nm and 0 – 6 nm, respectively. The movie is played at 7 frames/s.

**Supplementary Movie 4**

Height conversion of pro-Monalysin upon trypsin treatment. During trypsin injection, the AFM images were lost for a while. The scanning area was 200 × 200 nm^2^ with 100 × 100 pixels and the imaging rate was 330 ms/frame. The height scale is 0 – 15 nm. The movie is played at 15 frames/s.

**Supplementary Movie 5**

Pro-Monalysin under different tapping forces. *A_sp_/A_0_* was set to 0.95 for (a), 0.70 for (b), 0.60 for (c) and 0.50 for (d), respectively. The scanning area was 200 × 200 nm^2^ with 100 × 100 pixels and the imaging rate was 330 ms/frame. The height scale is 0 – 15 nm. The movie is played at 12 frames/s.

**Supplementary Movie 6**

Active-Monalysin under a strong tapping force. *A_sp_/A_0_* was set to 0.60. The scanning area was 200 × 200 nm^2^ with 100 × 100 pixels and the imaging rate was 330 ms/frame. The height scale is 0 – 15 nm. The movie is played at 12 frames/s.

**Supplementary Movie 7**

Pro-Monalysin on mica surface in the low salt buffer. The scanning area was 40 × 40 nm^2^ with 200 × 200 pixels and the imaging rate was 500 ms/frame. The height scale is 0 – 15 nm. The movie is played at 4 frames/s.

**Supplementary Movie 8**

Insertion of Active-Monalysin into lipid membrane formed on the PDMS surface. The scanning area was 150 × 150 nm^2^ with 80 × 80 pixels and the imaging rate was 250 ms/frame. The height scale is 0 – 12 nm. The movie is played at 20 frames/s.

**Supplementary Movie 9**

High-magnification view of active-Monalysin inserted into lipid membrane formed on PDMS surface. The scanning area was 30 × 30 nm^2^ with 150 × 150 pixels and the imaging rate was 330 ms/frame. The height scale is 0 – 7 nm. The movie is played at 6 frames/s.

**Supplementary Movie 10**

Rupture of a liposome on which the active-Monalysin are inserted. The scanning area was 200 × 200 nm^2^ with 80 × 80 pixels and the imaging rate was 200 ms/frame. The height scale is 0 – 15 nm. The movie is played at 10 frames/s.
